# Synthesis, Antiviral Bioactivity of Novel 4-Thioquinazoline Derivatives Containing Chalcone Moiety

**DOI:** 10.3390/molecules200711861

**Published:** 2015-06-29

**Authors:** Zhihua Wan, Deyu Hu, Pei Li, Dandan Xie, Xiuhai Gan

**Affiliations:** State Key Laboratory Breeding Base of Green Pesticide and Agricultural Bioengineering, Key Laboratory of Green Pesticide and Agricultural Bioengineering, Ministry of Education, Guizhou University, Huaxi District, Guiyang 550025, Guizhou Province, China; E-Mails: wzhuacool@163.com (Z.W.); pl19890627@126.com (P.L.); xddxed@163.com (D.X.); gxh200719@163.com (X.G.)

**Keywords:** 4-thioquinazoline derivatives, chalcone moiety, synthesis, antiviral activity, TMV, 3D-QASR, MST

## Abstract

A series of novel 4-thioquinazoline derivatives containing chalcone moiety were designed, synthesized and systematically evaluated for their antiviral activity against TMV. The bioassay results showed that most of these compounds exhibited moderate to good anti-TMV activity. In particular, compounds **M_2_** and **M_6_** possessed appreciable protection activities against TMV *in vivo*, with 50% effective concentration (EC_50_) values of 138.1 and 154.8 μg/mL, respectively, which were superior to that of Ribavirin (436.0 μg/mL). The results indicated that chalcone derivatives containing 4-thioquinazoline moiety could effectively control TMV. Meanwhile, the structure-activity relationship (SAR) of the target compounds, studied using the three-dimensional quantitative structure-activity relationship (3D-QSAR) method of comparative molecular field analysis (CoMFA) based on the protection activities against TMV, demonstrated that the CoMFA model exhibited good predictive ability with the cross-validated *q*^2^ and non-cross-validated *r*^2^ values of 0.674 and 0.993, respectively. Meanwhile, the microscale thermophoresis (MST) experimental showed that the compound **M_6_** may interaction with the tobacco mosaic virus coat protein (TMV CP).

## 1. Introduction

Tobacco mosaic virus (TMV) is a positive-sense single stranded RNA virus that infects members of nine plant families and at least 125 species, including tobacco, tomato, pepper, cucumbers, and a number of ornamental flowers. Once infected with TMV, leaves tend to become mosaic, yellow, necrosis, and ruguse, which would affect crop production and quality, and caused $100 million in economic losses in worldwide [1]. However, there are no effective antiviral agents for controlling TMV. Therefore, it is a challenge to the development of novel, potent, and structurally concise antiviral agents.

4-Thioquinazoline and their derivatives, an important class of heterocyclic compounds, have a wide range of biological properties [2], including antibacterial [3,4], antifungal [5,6,7], and anticancer [8] activities. Over the past few years, the synthesis and study of bioactivity of 4-thioquinazoline derivatives have attracted considerable attention. In our previous study, we have designed and synthesized a series of *S*-substituted 6-fluoro-4-alkyl(aryl)thioquinazoline derivatives and 6-bromo-4-alkylthioquinazoline derivatives which exhibited good antifungal activities [6,7].

Chalcones, belonging to the flavonoid family and obtained from *Carthamus tinctorius* first, possessed a broad spectrum of biological activities, including antibacterial [9,10,11,12], antifungal [13], anti-Alzheimer’s disease [14], anticancer [15], antitrichomonal [16], and anti-trypanosomacruzi [17] activities. It is reported that Verma *et al.*, had reported a series of chalcones derivatives, which were used to control plant viruses [18]. Meanwhile, some studies also demonstrated that chalcone derivatives could better control TMV [19], PVX [20], and ToRSV [21,22], respectively. Moreover, in our previous study, we have reported a series of chalcone derivatives with better bioactivities against TMV and CMV [23].

Tobacco mosaic virus coat protein (TMV CP) assembly systems include helix and four-layer aggregate disk systems. The helix forms of TMV CP mainly exist in the presence of TMV RNA. In these helix forms, TMV CP has an important role in the self-assembly of TMV through an initial RNA recognition reaction that triggers the assembly, which is believed to be necessary for virus assembly initiation and elongation. The four-layer aggregate disk forms consisting of 34 subunit aggregates of TMV CP are crystallized as a dimer of bilayer disks with 17 subunits per layer in the absence of TMV RNA [24].

In this work, a series of novel 4-thioquinazoline derivatives containing chalcone moiety were designed, synthesized and systematically evaluated their antiviral activities against TMV. Biological results showed that some of the title compounds displayed moderate to good antiviral activity. Among the title compounds, compounds **M_2_** and **M_6_** showed appreciable protection activities against TMV *in vivo*, which were better than that of the commercial agricultural antiviral agent Ribavirin. In addition, the structure-activity relationship (SAR) of the compounds was also discussed using comparative molecular field analysis (CoMFA) of the three-dimensional quantitative structure-activity relationship (3D-QSAR) method based on the protection activities against TMV. The microscale thermophoresis (MST) experimental showed that the compound **M_6_** may interactions with the TMV CP. To the best of our knowledge, this is the first report on the synthesis, antiviral activity, 3D-QSAR, and interaction study of 4-thioquinazoline derivatives containing chalcone moiety.

## 2. Results and Discussion

### 2.1. Chemistry

**Scheme 1:**
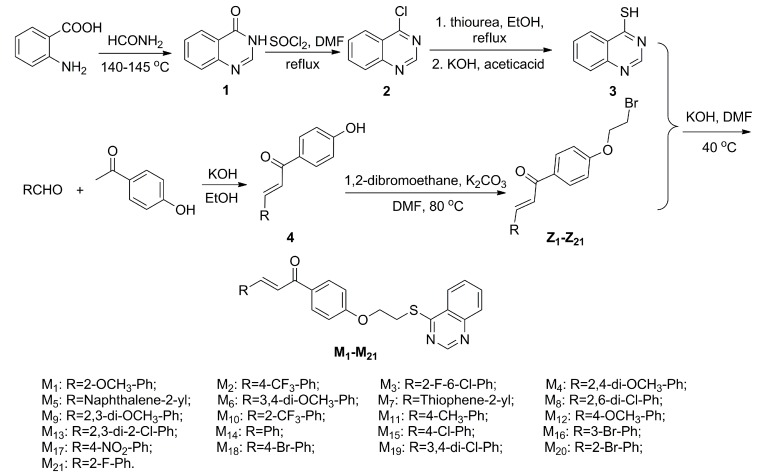
Synthetic route of the target compounds.

The summary of the synthetic route designed for the 4-thioquinazoline derivatives containing chalcone moiety were shown in Scheme 1. Substituted 2-aminobenzoicacid, using as the starting materials, was reacted with formamide for 4.5 h at 140 to 145 °C to obtain the intermediate 4(*3H*)-quinazolinone (**1**). Substituted 4-chloroquinazoline (**2**) was prepared by chlorination reaction with SOCl_2_ [8]. Then, using ethanol as the solvent, nucleophilic substitution reaction of **2** and carbamimidothioic acid, transformed from thiourea via keto-enol tautomerism equilibrium, was reacted 8 h at 87 °C to obtain the intermediate quinazolin-4-yl carbamimidothioate, then quinazolin-4-yl carbamimidothioate was reacted with potassium hydrate, and adjusted pH value to 7 by acetic acid to get the key intermediate quinazoline-4-thiol (**3**) [25,26]. Intermediates **4** was obtained via condensation of the 1-(4-hydroxyphenyl)ethanone with substituted aldehydes. Then, intermediates **Z_1_**–**Z_21_** were obtained via nucleophilic substitution reaction of **4** and 1,2-dibromoethane in DMF for 8 h at 80 °C. Finally, the target compounds **M_1_**–**M_21_** were synthesized by the etherification reaction of intermediates **3** and **Z** with KOH in DMF at 40 °C for 8 to 10 h.

The structures of all the title compounds confirmed through IR, ^1^H-NMR, ^13^C-NMR spectral and elemental analyses. The IR spectra of the title compounds **M_1_**–**M_21_** exhibited characteristic absorption bands at 1690–1640 cm^−1^, which indicated the presence of C=O. The stretching frequency at 1620–1600 cm^−1^ was assigned to C=N vibrations. ^1^H-NMR indicated that all of the phenyl protons showed multiplets at 8.27–6.93 ppm. The main characteristic of the ^1^H-NMR spectra for the target compounds was the presence of a high-frequency downfield singlet δ_H_ 9.00–9.02 for Qu-2-H. And –O–CH_2_– and –S–CH_2_– showed a triplet at 4.40–4.45 ppm and 3.80–3.84 ppm, respectively. Moreover, the chemical shifts shown at nearly 188.40–189.40, 66.50–66.60, and 28.22–28.33 ppm in ^13^C-NMR were also confirmed the presence of C=O, –OCH_2_–, and –SCH_2_–, respectively.

### 2.2. Antiviral Activity Screening of Title Compounds against TMV in Vivo

The antiviral activities of the target compounds **M_1_**–**M_21_** against TMV were evaluated by the half-leaf method [27] and the results were summarized in Table 1. Most of the title compounds generally exhibited good antiviral activity against TMV *in vivo*. The results of the preliminary bioassays indicated that compounds **M_1_**, **M_2_**, and **M_6_** exhibited better curative activities against TMV at 500 μg/mL, with the values of 52.5% ± 5.2%, 47.7% ± 4.3%, and 48.3% ± 3.3%, respectively. Which were better than that of Ribavirin (37.9% ± 1.9%). Compounds **M_2_** and **M_6_** exhibited significant protection activities against TMV at 500 μg/mL, with the values of 68.6% ± 7.7% and 72.3% ± 5.2%, respectively, which were even better than that of Ribavirin (51.8% ± 2.3%). The inactivation activities of compounds **M_2_**, **M_6_**, **M_8_**, **M_10_**, and **M_14_** at 500 μg/mL were 82.1% ± 4.1%, 83.2% ± 4.4%, 85.6% ± 2.6%, and 85.1% ± 3.6%, respectively, which were better than that of Ribavirin (72.9% ± 2.4%).

**Table 1:** Antiviral activities of the test compounds against tobacco mosaic virus (TMV) *in vivo* at 500 μg/mL.

Compd.	Curative Activity (%) ^*a*^	Protection Activity (%) ^*a*^	Inactivation Activity (%) ^*a*^
**M_1_**	52.5 ± 5.2	60.7 ± 4.3	65.4 ± 2.9
**M_2_**	47.7 ± 4.3	68.6 ± 7.7	82.1 ± 4.1
**M_3_**	42.1 ± 6.1	57.4 ± 2.4	63.4 ± 6.8
**M_4_**	45.3 ± 2.3	50.2 ± 2.2	83.2 ± 4.4
**M_5_**	29.8 ± 4.2	59.3 ± 5.9	63.9 ± 6.1
**M_6_**	48.3 ± 3.3	72.3 ± 5.2	79.6 ± 3.4
**M_7_**	43.2 ± 6.7	56.3 ± 4.6	66.1 ± 5.0
**M_8_**	45.1 ± 6.6	51.1 ± 4.2	85.6 ± 2.6
**M_9_**	43.2 ± 3.5	49.4 ± 5.2	75.2 ± 3.8
**M_10_**	46.5 ± 5.6	62.2 ± 2.1	85.1 ± 3.6
**M_11_**	42.4 ± 4.3	57.3 ± 3.6	71.5 ± 5.1
**M_12_**	38.4 ± 4.5	57.6 ± 1.2	66.0 ± 2.8
**M_13_**	46.2 ± 5.8	51.8 ± 5.5	70.8 ± 2.7
**M_14_**	46.5 ± 3.7	62.3 ± 6.2	83.6 ± 2.3
**M_15_**	38.7 ± 6.2	52.8 ± 4.7	62.9 ± 6.0
**M_16_**	38.5 ± 4.3	48.3 ± 5.2	57.3 ± 3.5
**M_17_**	46.3 ± 4.3	57.4 ± 5.1	61.5 ± 3.2
**M_18_**	45.7 ± 3.8	61.3 ± 4.5	66.4 ± 4.2
**M_19_**	41.2 ± 6.2	60.3 ± 3.5	65.6 ± 4.7
**M_20_**	41.2 ± 3.2	47.3 ± 4.1	63.6 ± 4.3
**M_21_**	45.2 ± 6.2	55.6 ± 6.1	60.3 ± 5.9
Ribavirin	37.9 ± 1.9	51.8 ± 2.3	72.9 ± 2.4

^*a*^: Average of three replicates.

Based on the previous bioassays, the 50% effective concentration (EC_50_) values of protection activities against TMV of the title compounds were tested and presented in Table 2. As indicated in Table 2, most of the target compounds showed good anti-TMV activities. Compounds **M_1_**, **M_2_**, **M_4_**, **M_6_**, **M_10_**, **M_11_**, **M_12_**, **M_14_**, **M_15_**, **M_18_**, and **M_19_** exhibited higher protection activity than Ribavirin (436.0 ± 4.3 μg/mL), with the EC_50_ values range from 138.1 ± 3.4 to 274.3 ± 6.2 μg/mL. Especially, compounds **M_2_** and **M_6_** exhibited the best protection activity against TMV, with the EC_50_ values of 156.4 ± 4.1 and 138.1 ± 3.4 μg/mL, respectively, which were better than that of Ribavirin (436.0 ± 4.3 μg/mL). Compounds **M_5_**, **M_7_**, **M_13_**, **M_16_**, and **M_21_** exhibited moderate protection activity against TMV, with EC_50_ values of 355.9 ± 3.5, 406.9 ± 5.2, 424.0 ± 1.9, 345.1 ± 3.6, and 342.5 ± 4.3 μg/mL. This finding suggests that these compounds may be potential lead structures for the discovery of new antiviral agents.

**Table 2:** Actual and predicted protection activities against TMV.

Compd.	EC_50_ (μg/mL) ^a^	Actual pEC_50_ (μM) ^b^	Predicted pEC_50_ (μM) ^b^	Residual
**M_1_**	255.2 ± 3.2	3.239	3.249	−0.010
**M_2_**	156.4 ± 4.1	3.487	3.494	−0.007
**M_3_**	444.4 ± 2.6	3.020	3.015	0.005
**M_4_**	208.1 ± 4.2	3.356	3.351	0.005
**M_5_**	355.9 ± 3.5	3.114	3.066	0.048
**M_6_**	138.1 ± 3.4	3.534	3.531	0.003
* M_7_	406.9 ± 5.2	3.012	3.069	−0.057
**M_8_**	442.1 ± 4.3	3.037	3.042	−0.005
**M_9_**	486.3 ± 6.3	2.988	2.994	−0.006
**M_10_**	204.6 ± 3.6	3.371	3.373	−0.002
**M_11_**	218.1 ± 5.2	3.291	3.286	0.005
* M_12_	314.6 ± 2.6	3.148	3.194	−0.046
**M_13_**	424.0 ± 1.9	3.055	3.051	0.004
**M_14_**	319.5 ± 3.7	3.111	3.083	0.028
**M_15_**	274.3 ± 6.2	3.212	3.257	−0.045
**M_16_**	345.1 ± 3.6	3.153	3.057	0.096
**M_17_**	198.2 ± 5.2	3.363	3.349	0.014
**M_18_**	246.6 ± 3.2	3.299	3.268	0.031
* M_19_	211.2 ± 4.2	3.358	3.295	0.063
**M_20_**	488.2 ± 3.5	2.945	2.942	0.003
**M_21_**	342.5 ± 4.3	3.099	3.110	−0.011
Ribavirin	436.0 ± 4.3	/	/	/

^a^: Average of three replicates; ^b^: pEC_50_ = −lg (EC_50_); *: Samples of the testing set.

### 2.3. Antiviral Activity and Structure Activity Relationship against TMV

As an extension of this approach, the structure-activity relationships were deduced on the basis of activity values in Table 1 and Table 2. Some of the compounds showed potency against TMV. When the R were 2-CH_3_-Ph (**M_1_**), 4-CF_3_-Ph (**M_2_**), 3,4-di-OCH_3_-Ph (**M_6_**), and 2-CF_3_-Ph (**M_10_**) groups, the corresponding target compounds exhibited good curative activity. When R group was substituted with fused ring, it was disfavored for the anti-TMV following the order of **M_5_** (Naphthalene-2-yl) < **M_7_** (Thiophene-2-yl). And, when the R were 4-CF_3_-Ph (**M_2_**) and 3,4-di-OCH_3_-Ph (**M_6_**) groups, the corresponding compounds exhibited good protection activities against TMV which was surpassed that of Ribavirin. However, compared with **M_3_** (2-Cl-6-F-Ph), **M_8_** (2,6-di-Cl-Ph), **M_15_** (4-Cl-Ph), and **M_19_** (3,4-di-Cl-Ph), we found that the one who has dichlorophenyl substituent groups could increase the activity against TMV (**M_8_** and **M_15_** > **M_3_** and **M_13_**). Furthermore, the presence of 4-(trifluoromethyl)phenyl (**M_2_**), 3,4-dimethoxypheny (**M_6_**), and 3,4-dichlorophenyl (**M_19_**) groups in a compound effectively improved the antiviral activity of the compound more than that of other groups. Moreover, compared with **M_2_** (4-CF_3_-Ph), **M_10_** (2-CF_3_-Ph), **M_3_** (2-Cl-6-F-Ph), **M_20_** (2-F-Ph), and **M_21_** (4-F-Ph), we found that the one who has trifluoromethyl substituent groups increases in the activity against TMV (**M_2_** and **M_10_** > **M_3_**, **M_20_** and **M_21_**).

### 2.4. 3D-QSAR Study

**Figure 1:**
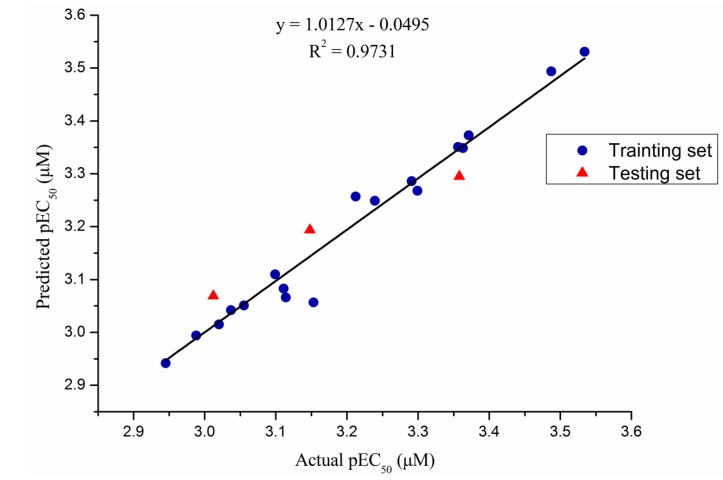
Plot of actual predicted activities for training set and test set based on the comparative molecular field analysis (CoMFA) model.

In order to analyze the SAR base on the protection activity against TMV, the CoMFA of 3D-QSAR model [28] with a total of twenty-one target compounds were developed by using Sybyl 7.3 software [29] from Tripos Inc. (St. Louis, MO, USA). Predicted pEC_50_ [30] values of compounds in both the training and testing sets were presented together with their actual pEC_50_ values in Table 2, and correlations between predicted and actual pEC_50_ in CoMFA model were presented in Figure 1. Overall, predicted EC_50_ values were very close to the corresponding actual values for compounds in both the training and testing sets. The mostly linear correlations in Figure 1 demonstrated high predictive power of the CoMFA model. Meanwhile, as shown in Table 3, the non-cross-validated PLS analysis was repeated with the optimum number of components, as determined by the cross-validated analysis. To obtain statistical confidence limits, the non-cross-validated analysis was repeated, which yielded an *r*^2^ value of 0.993, and the *q*^2^ value of highly predictive CoMFA was 0.674 with 8 ONC, which suggested that the model has good predictive ability (*r*^2^ > 0.9, *q*^2^ > 0.5). Meanwhile, as shown in Table 3, the SEE was 0.020 and the *F* value was 161.503, respectively. The relative contributions to bioactivity from steric and electrostatic fields in the CoMFA model were 0.478 and 0.522, respectively, suggesting that bioactivity was mainly determined by electrostatic interactions.

**Table 3:** Statistical parameters of the CoMFA model.

Statistical Parameter	CoMFA
*q*^2 a^	0.674
ONC ^b^	8
*r*^2 c^	0.993
SEE ^d^	0.020
*F* ^e^	161.503
Steric ^f^	0.478
Electrostatic ^g^	0.522

^a^: Cross-validated correlation; ^b^: Optimum number of components; ^c^: Non-cross-validated correlation; ^d^: Standard error of estimate; ^e^: *F* value; ^f^: Stericfield contribution; ^g^: Electrostatic field electrostatic.

**Figure 2:**
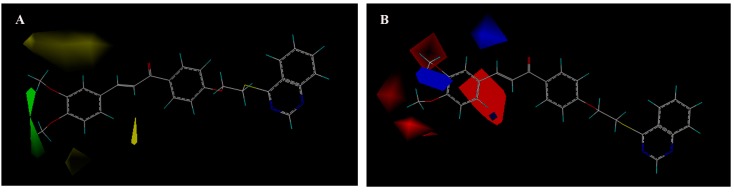
CoMFA contour map for the steric (**A**) and electrostatic (**B**) component.

The CoMFA contour map of the steric was shown in Figure 2A. Green contours in the CoMFA steric field indicated regions where bulky groups would increase activity, whereas yellow contours indicated regions where bulky groups would decrease activity. As shown in Figure 2A, a green contour around 3- and 4-positions of aromatic ring suggested that anti-TMV activity increases with bulky substituents in the order of **M_6_** (3,4-di-OCH_3_-Ph, 1.898 Å^3^) > **M_9_** (3,4-di-Cl-Ph, 1.707 Å^3^), and **M_2_** (4-CF_3_-Ph, 1.616 Å^3^) > **M_12_** (4-OCH_3_-Ph, 1.586 Å^3^). A larger yellow contour around 2- and 3-positions of aromatic ring showed that compounds with bulky groups at this position exhibited lower anti-TMV activity in the order of **M_13_** (2,3-di-Cl-Ph, 1.725 Å^3^) > **M_9_** (2,3-di-OCH_3_-Ph, 1.494 Å^3^).

The CoMFA contour map of the electrostatic was shown in Figure 2B. Blue contours in the CoMFA electrostatic field indicated regions where electron withdrawing groups would increase activity and red contours indicated regions where electron-donating groups would increase activity. As shown in Figure 2B, a red contour and a blue contour around 3- and 4-position of aromatic ring, which indicated that suitable groups in the region could increase the activities follow the activity order of **M_15_** (4-Cl-Ph) > **M_12_** (4-OCH_3_-Ph) > **M_21_** (4-F-Ph). Meanwhile, a blue contour around 2-position of aromatic ring showed that the electron withdrawing groups were favored at improvement of activity in the order of **M_10_** (2-CF_3_-Ph) > **M_1_** (2-OCH_3_-Ph).

3D-QASR results indicated that introduction of small and electron withdrawing groups at 2-position of aromatic ring could largely improve the activity. Bulky groups at 3- and 4-position of the aromatic ring played a favorable role in improving the activity.

### 2.5. Binding Sites of M_6_, M_15_ and Ribavirin to TMV CP

**Figure 3:**
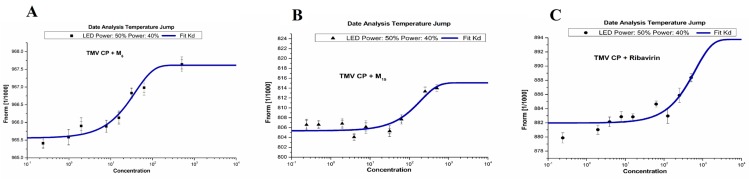
Microscale thermophoresis (MST) of **M_6_** (**A**); **M_15_** (**B**); and Ribavirin (**C**).

In order to study the interactions between target compounds and TMV CP, the MST analysis method was used. The MST results indicated that the compounds **M_6_**, **M_15_**, and Ribavirin binding to TMV CP protein yielded *K*_d_ values of 31.1 ± 1.83, 346 ± 23.9, and 511 ± 33.1 μM, respectively (Figure 3). As predicted in MST, **M_6_** shares, indeed, moderate affinity, in the contrary to **M_15_** and Ribavirin, which share weak affinity. The results showed that the combining capacity in the order of **M_6_** > **M_15_** > Ribavirin is consistent with the trend of antiviral activity screening. The experimental results showed that the compound **M_6_** may interactions with the TMV CP. As shown in the Figure 3 and Table 4, bulky groups at 3- and 4-position of aromatic ring played a favorable role in improving the activity. The bioactivity was mainly determined by electrostatic interactions. As shown in Figure 3 and Table 4, when bulky group at 3- and 4-position of aromatic ring (3,4-di-OCH_3_-Ph), the corresponding compound (**M_6_**) played a stronger combining capacity, with the *K*_d_ values of 31.1 ± 1.83 μM, compared with that of compound **M_15_** (346 ± 23.9 μM), which was substituted with smaller group at 4-position of aromatic ring (4-Cl-Ph).

**Table 4:** The dissociation constant of **M_6_**, **M_15_**, and Ribavirin with TMV- coat protein (CP).

Compd.	*K*_d_ (μM)
**M_6_**	31.1 ± 1.83
**M_15_**	346 ± 23.9
Ribavirin	511 ± 33.1

## 3. Experimental Section

### 3.1. Instruments

^1^H-NMR and ^13^C-NMR spectra were obtained at 500 MHz using a JEOL-ECX500 NMR spectrometer at room temperature using tetramethylsilane as an internal standard (solvent CDCl_3_). Elemental analysis was performed on an Elementar Vario-III CHN analyzer. The melting points of the products were determined under an XT-4 binocular microscope (Beijing Tech. Instrument Co., Beijing, China) and left untouched. Analytical thin-layer chromatography (TLC) was conducted on a silica gel GF254 (400 mesh). Column chromatographic operations were performed on silica gel (200–300 mesh). Tobacco seeds were provided by the Guizhou Institute of Tobacco.

### 3.2. Chemistry

#### 3.2.1. General Procedure for Preparation of Intermediates **3**

Quinazolin-4(3*H*)-one (**1**) and4-chloroquiazoline (**2**) were prepared according to a previously described method [8]. 4-thioquinazoline (**3**) was gained by 4-chloroquiazoline with thiourea at reflux temperature for 8 h. The data for intermediates quinazolin-4(3*H*)-one (**1**), 4-chloroquiazoline (**2**), and 4-thioquinazoline (**3**) can be found in the previously references [8,25].

#### 3.2.2. General Procedure for Preparation of Intermediates **Z**_**1**–**21**_

A mixture of **4** (4.95 mmol) and K_2_CO_3_ (9.99 mmol) in DMF (10 mL) was stirred at 80 °C for 1 h. Then, 1,2-dibromoethane (14.84 mmol) was added dropwise. The mixture was stirred until the TLC showed the reaction finished. The reaction mixture was poured into water (20 mL). The mixture was extracted with EtOAc (3 × 10 mL) and the combined organic layers were dried over anhydrous Na_2_SO_4_ and concentrated under reduced pressure. The crude product was purified by column chromatography over silicagel by using petroleum ether and ethyl acetate (*v*/*v* = 2:1) as eluent to give **Z**_**1**–**21**_ as a solid.

#### 3.2.3. General Procedure for Preparation of Title Compounds (**M_1_**–**M_21_**)

The target compounds **M_1_**–**M_21_** were synthesized as schematized in Scheme 1. A 25 mL round-bottomed flask equipped with a magnetic stirrer was charged with intermediates **3** (0.74 mmol), KOH (0.89 mmol), DMF (5 mL). The flask was stirred at 40 °C for 1 h, and then **Z** (0.59 mmol) in DMF (2 mL) was dropwise into the flask. The resulting mixture was stirred at 40 °C for 8 to 10 h. TLC monitored the progress of the reaction. Upon completion of the reaction (as indicated by TLC), the reaction mixture was poured into saturated brine, the solid was filtered off and then dissolved with dichloromethane and washed by 10% KOH. The organic fraction was evaporated under reduced pressure. The solid was recrystallized from ethyl acetate/petroleum ether (3:1, *v*/*v*) to obtain the title compounds **M_1_**–**M_21_** with the yields from 56% to 78%. The physical characteristics, IR, ^1^H-NMR, ^13^C-NMR, and elemental analysis data, for all the synthesized compounds were reported in Supplementary data and the representative data of **M_6_** were shown below.

*(E)-3-(3*,*4-Dimethoxyphenyl)-1-(4-(2-(quinazolin-4-ylthio)ethoxy)phenyl)prop-2-en-1-one* (**M_6_**). Yellow solid; m.p. 151.2–153.1 °C; yield, 72.5%; IR (KBr, cm^−1^) *ν*: 3023.5–3068.8 (C-H of benzene), 2832.5 (–OCH_3_), 1654.0 (C=N), 1604.9 (C=O), 1490.0–1562.4 (C=C and benzene and Qu-ring), 1328.0 (C–N), 1260.5 (C–O); 1172.7 (–O–CH_3_); ^1^H-NMR (500 MHz, CDCl_3_) δ: 9.01 (s, 1H, Qu-2-**H**), 8.10 (d, *J* = 7.45 Hz, 1H, Qu-8-**H**), 8.04 (d, *J* = 9.15 Hz, 2H, CO–Ph-2,6-**H**), 7.98 (d, *J* = 8.55 Hz, 1H, Qu-5-**H**), 7.86 (t, *J*_1_ = 8.60 Hz, *J*_2_ = 6.85 Hz, 1H, Qu-7-**H**), 7.77 (d, 1H, *J* = 15.45 Hz, Ar–C**H**), 7.60 (t, *J*_1_ = 7.40 Hz, *J*_2_ = 6.90 Hz, 1H, Qu-6-**H**), 7.41 (d, *J* = 15.45 Hz, 1H, Ph–CO=C**H**), 7.23 (dd, *J*_1_ = 8.50 Hz, *J*_2_ = 1.70 Hz, 1H, Ar-2-**H**), 7.15 (d, *J* = 1.75 Hz, 1H, Ar-6-**H**), 7.07 (d, *J* = 8.60 Hz, 2H, CO–Ph-3,5-**H**), 6.90 (d, *J* = 8.50 Hz, 1H, Ar-5-**H**), 4.42 (t, *J*_1_ = 6.60 Hz, *J_2_* = 7.40 Hz, 2H, –OC**H**_2_–), 3.95 (s, 3H, –C**H**_3_), 3.92 (s, 3H, –C**H**_3_), 3.81 (t, *J*_1_ = 7.35 Hz, *J*_2_ = 6.30 Hz, 2H, –SCH_2_–); ^13^C-NMR (125 MHz, CDCl_3_) δ: 188.71, 170.25, 162.13, 153.41, 151.26, 149.20, 148.09, 144.24, 133.92, 131.62, 130.76, 128.90, 128.01, 127.54, 123.89, 123.77, 122.98, 119.75, 114.41, 111.10, 110.07, 66.46, 55.99, 55.97, 28.22; Anal. Calcd for C_27_H_22_N_2_O_4_: C, 68.62; H, 5.12; N, 5.93; Found: C, 68.30; H, 5.11; N, 5.91.

### 3.3. Antiviral Biological Assay

#### 3.3.1. Purification of TMV

Using Gooding’s method [21], the upper leaves of *N. tabacum cv. K326* inoculated with TMV were selected, ground in phosphate buffer, and then filtered through a double-layer pledget. The filtrate was centrifuged at 10,000 *g*, treated twice with PEG, and then centrifuged again. The entire experiment was conducted at 4 °C. Absorbance values were estimated at 260 nm using an ultraviolet spectrophotometer.

(1) $$\text{virus}\;\text{concn}=\left(A_{260}\times \text{diluton}\;\text{ratio}\right)/E_{1\;cm}^{0.1\%,\;260\;nm}$$

#### 3.3.2. Curative Effects of the Target Compounds against TMV *in Vivo*

Growing 5–6-leaf stage *Nicotiana tabacum* L. tobaccos were selected. TMV (concentration of 6 × 10^−3^ mg/mL) was dipped and inoculated using a brush on the whole leaves, which were previously then dried. The compound solution was smeared on the left side of the leaves, scattered with silicon carbide. The leaves were then washed with water after inoculation for 0.5 h and the solvent was smeared on the right side for control. All plants were cultivated in an incubator at a temperature of 23 ± 1 °C and an illumination of 10,000 Lux. The number of local lesions was counted and recorded 3 to 4 days after inoculation. Three repetitions were conducted for each compound [27].

#### 3.3.3. Protection Effects of the Target Compounds against TMV *in Vivo*

The compound solution was smeared on the left side, whereas the solvent was smeared on the right side of *Nicotiana tabacum* L. leaves of the same age to serve as the control. The leaves were inoculated with the virus after 12 h. A brush was dipped in 6 × 10^−3^ mg/mL TMV to inoculate the leaves which were previously scattered with silicon carbide. Subsequently, the leaves were washed with water and rubbed softly along the nervature once or twice. All plants were cultivated in an incubator at a temperature of 23 ± 1 °C and an illumination of 10,000 Lux. The number of local lesions was counted and recorded 3 to 4 days after inoculation. Three repetitions were conducted for each compound [27].

#### 3.3.4. Inactivation Effects of the Target Compounds against TMV *in Vivo*

The virus was inhibited by mixing with the compound solution at the same volume for 30 min. The mixture was then inoculated on *Nicotiana tabacum* L. leaves, and the right side of the leaves was inoculated with solvent and virus mixture for control. All of the leaves were previously scattered with silicon carbide. All plants were cultivated in an incubator at a temperature of 23 ± 1 °C and an illumination of 10,000 Lux. The number of local lesions was counted and recorded 3 to 4 days after inoculation. Three repetitions were conducted for each compound [27].

The inhibitory rate of the compounds was calculated according to the following formula (“av” denotes average):

(2) Inhibition rate (%) = [(av local lesion No. of control (not treated with compd.) − av local lesion No. smeared with drugs)/av local lesion No. of control (not treated with compd.)] × 100%

### 3.4. 3D-QSAR Study

The protection activity used in study was expressed as pEC_50_ listed in Table 2, 18 molecules of total compounds were randomly chosen as the training set for CoMFA and the other three compounds (asterisk labeled) were used as the testing set.

#### 3.4.1. Molecular Modeling and Alignment

Molecular modeling, CoMFA analysis was performed using Sybyl 7.3 (Tripos Inc., St. Louis, MO, USA) software. The 3D structures of all molecules were built using the “Sketch Molecule” function in Sybyl. Initial optimization of the structures were carried out using the Gasteiger-Hückel charge, Tripos force field, and Powell conjugate gradient algorithm with a convergence criterion of 0.005 kcal/mol·Å [28]. The 3D structures of the 21 molecules were aligned on a common template molecule with **M_6_** for the CoMFA modeling study.

#### 3.4.2. Partial Least-Squares Analysis

The partial least squares (PLS) analysis was used to derive the 3D-QSAR models. In which, molecules were placed in a rectangular grid, the steric and electrostatic fields were calculated using a volume-dependent lattice with a 2.0 Å grid spacing [29], and the CoMFA descriptors was used as the independent variables, and the experimental pEC_50_ values were presented as the dependent variables. Then, 3D-QSAR analysis was carried out using the PLS technique. The cross-validation and the ONC were used to evaluate the performance of the models, ONC was determined with the highest cross-validated *q*^2^ [30,31]. Then, the non-cross-validated correlation coefficient *r*^2^ value, standard error of estimate (SEE), and *F* value and standard error were calculated according to the definitions in Sybyl 7.3 package, and as factors for estimating. The contour maps and standard deviations values of CoMFA was generated by the PLS coefficients.

### 3.5. MST Studies

TMV CP was purified according to a previously described method [24]. A range of concentrations of the required compounds (range from 0.1 to 2 mM) were incubated with 0.1 mM of purified recombinant TMV CP for 5 min with the Monolith NT Protein Labeling Kit Red (Nano Temper Technologies, München, Germany) in assay buffer (10 mM Tris/HCl and 100 mM sodium chloride, pH 7.4). The sample was loaded into the NanoTemper glass capillaries and microthermophoresis carried out using 50% LED power and 40% MST. The *K*_d_ values were calculated from the duplicate reads of three separate experiments using the mass action equation in the NanoTemper software [32].

## 4. Conclusions

In summary, a series of 4-thioquinazoline derivatives containing chalcone moiety were prepared and evaluated for their antiviral activities against TMV using half-leaf method *in vivo*. Bioassay results indicated that compounds **M_2_** and **M_6_** possessed appreciable protection activities against TMV *in vivo*, with the EC_50_ values of 156.4 and 138.1 μg/mL, respectively, which were superior to that of Ribavirin (436.0 μg/mL). Meanwhile, the CoMFA model was generated base on the protection activities against TMV and exhibited good predictive abilities with the cross-validated *q*^2^ and non-cross-validated *r*^2^ values of 0.674 and 0.993, respectively. The MST experimental showed that the compound **M_6_** may interaction with the TMV CP. The model provided a practical tool for the modification and optimization of 4-thioquinazoline derivatives containing chalcone moiety to further improve the antiviral activity.

## Supplementary Materials

Supplementary materials can be accessed at: http://www.mdpi.com/1420-3049/20/07/11861/s1.
