# Supplementary material for: Synthesis, Antiviral Bioactivity of Novel 4-Thioquinazoline Derivatives Containing Chalcone Moiety
Source: Molecules. 2015 Jun 29;20(7):11861–74. doi: 10.3390/molecules200711861 (PMC6332188; doi:10.3390/molecules200711861)
Supplement: Supplementary file 1 [file molecules-20-11861-s001.pdf]

Supplementary Materials

General Remarks

^{1}H-NMR and ^{13}C-NMR spectra were obtained at 500 MHz using a JEOL-ECX500 NMR spectrometer at room temperature using tetramethylsilane as an internal standard (solvent $CDCl_3$). Elemental analysis was performed on an Elementar Vario-III CHN analyzer. The melting points of the products were determined under an XT-4 binocular microscope (Beijing Tech Instrument Co., Beijing, China) and left untouched. Analytical thin-layer chromatography (TLC) was conducted on a silica gel GF254 (400 mesh). Column chromatographic operations were performed on silica gel (200–300 mesh). Tobacco seeds were provided by the Guizhou Institute of Tobacco.

General Procedure for Preparation of Intermediates 3

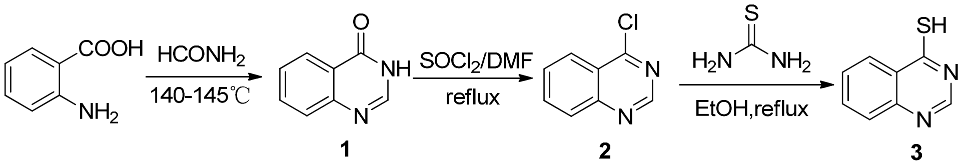

Quinazolin-4(3H)-one (**1**), and 4-chloroquiazoline (**2**) were prepared according to a previously described method [1]. 4-thioquinazoline (**3**) was gained by 4-chloroquiazoline with thiourea at reflux for 8 h.

The data for intermediate quinazolin-4(3*H*)-one (**1**) and 4-chloroquiazoline (**2**) can be found in the reference [1]. And 4-thioquinazoline (**3**) can be found in the reference [2].

General Procedure for Preparation of Intermediates $Z_{1–21}$

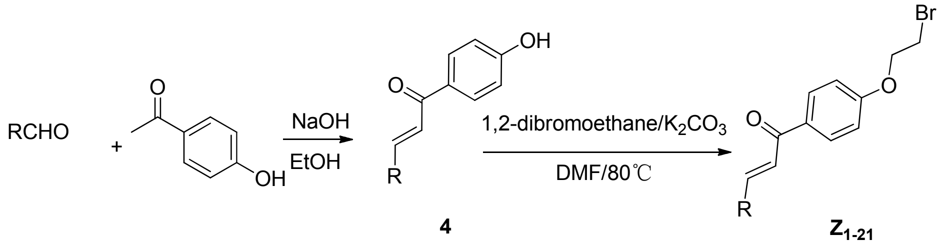

Intermediates $\mathbf{4_{a–u}}$ were prepared according to a previously described method [3]. Intermediate $\mathbf{Z_{1–21}}$ was synthesized by nucleophilic unimolecular with 1, 2-dibromoethane. A mixture of **4** (4.95 mmol) and K_2CO_3 (9.99 mmol) in DMF (10 mL) was stirred at 80 °C for 1 h. Then 1,2-dibromoethane (14.84 mmol) was added dropwise. The mixture was stirred until TLC showing the reaction was finished. The reaction mixture was poured into water (20 mL). The mixture was extracted with EtOAc (3 × 10 mL), combined organic layers were dried over anhydrous Na_2SO_4 and concentrated under reduced pressure. The crude product was purified by column chromatography over silicagel by using petroleum ether and ethyl acetate (*v*/*v* = 2:1) as eluent to give $\mathbf{Z_{1–21}}$ as a solid.

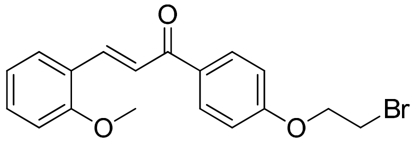

$\mathbf{Z_1}$

(E)-1-(4-(2-bromoethoxy)phenyl)-3-(2-methoxyphenyl)prop-2-en-1-one (**Z_1**). Yellow solid; m.p. 97.7–99.2 °C; yield, 57.6%; ^{1}H-NMR (500 MHz, $CDCl_3$) δ in ppm: 8.12 (d, *J* = 14.89 Hz, 1H, Ar–CH), 8.04 (d, *J* = 7.45 Hz, 2H, CO–Ph-2,6-H), 7.64–7.60 (m, 2H, Ar-6-H, Ph–CO=CH), 7.38 (t, J_1 = 7.45 Hz, J_2 = 8.02 Hz, 1H, Ar-4-H), 7.01–6.98 (m, 3H, Ar-5-H, CO–Ph-3,5-H), 6.95 (d, *J* = 8.02 Hz, 1H, Ar-3-H), 4.38 (t, J_1 = 6.30 Hz, J_2 = 4.58 Hz, 2H, $–OCH_2–$), 3.92 (s, 3H, $–OCH_3$), 3.68 (t, J_1 = 4.58 Hz, J_2 = 6.30 Hz, 2H, $–SCH_2–$); ^{13}C-NMR (125 MHz, $CDCl_3$) δ: 189.41, 161.72, 158.84, 139.87, 132.10, 131.70, 130.96, 129.29, 124.13, 122.69, 120.81, 114.43, 111.30, 67.92, 55.64, 28.77.

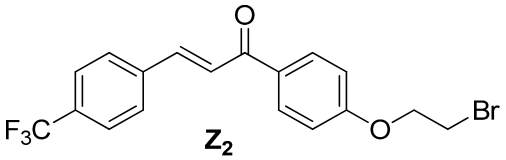

$\mathbf{Z_2}$

(E)-1-(4-(2-bromoethoxy)phenyl)-3-(4-(trifluoromethyl)phenyl)prop-2-en-1-one (**Z_2**). Yellow solid; m.p. 113.5–115.2 °C; yield, 56.7%; ^{1}H NMR (500 MHz, $CDCl_3$) δ in ppm: 8.06 (d, *J* = 8.59 Hz, 2H, CO–Ph-2,6-H), 7.81 (d, *J* = 16.04 Hz, 1H, Ar–CH), 7.74 (d, *J* = 8.02 Hz, 2H, Ar-2,6-H), 7.68 (d, *J* = 8.59 Hz, 2H, Ar-3,5-H), 7.61 (d, *J* = 16.04 Hz, 1H, Ph–CO=CH), 7.02 (d, *J* = 8.59 Hz, 2H, CO–Ph-3,5-H), 4.38 (t, J_1 = 5.73 Hz, J_2 = 6.30 Hz, 2H, $–OCH_2–$), 3.68 (t, J_1 = 6.30 Hz, J_2 = 5.73 Hz, 2H, $–SCH_2–$); ^{13}C-NMR (125 MHz, $CDCl_3$) δ: 188.24, 162.19, 142.22, 138.48, 131.70, 131.37, 131.08, 128.54, 126.00, 125.97, 124.03, 114.63, 67.97, 28.69.

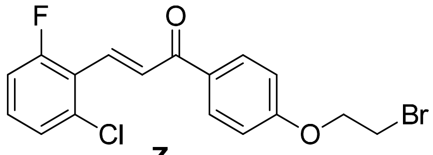

$\mathbf{Z_3}$

(E)-1-(4-(2-bromoethoxy)phenyl)-3-(2-chloro-6-fluorophenyl)prop-2-en-1-one (**Z_3**). Yellow solid; m.p. 132.1–133.6 °C; yield, 59.2%; ^{1}H-NMR (500 MHz, $CDCl_3$) δ in ppm: 8.05 (d, *J* = 9.16 Hz, 2H, CO–Ph-2,6-H), 8.05 (d, *J* = 15.46 Hz, 1H, Ar–CH), 7.82 (d, *J* = 16.04 Hz, 1H, Ph–CO=CH), 7.29–7.27 (m, 2H, Ar-3,4-H), 7.10–7.06 (m, 1H, Ar-5-H) 7.01 (d, *J* = 8.59 Hz, 2H, CO–Ph-3,5-H), 4.38 (t, J_1 = J_2 = 6.30 Hz, 2H, $–OCH_2–$), 3.67 (t, J_1 = 6.01 Hz, J_2 = 6.59 Hz, 2H, $–SCH_2–$); ^{13}C-NMR (125 MHz, $CDCl_3$) δ: 188.73, 162.11, 136.61, 133.97, 131.45, 131.60, 130.75, 128.61, 122.49, 115.05, 114.86, 114.58, 67.95, 28.67.

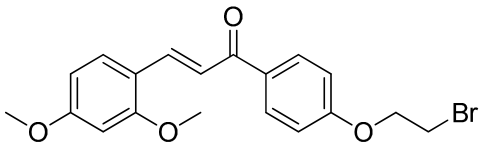

$\mathbf{Z_4}$

(E)-1-(4-(2-bromoethoxy)phenyl)-3-(2,4-dimethoxyphenyl)prop-2-en-1-one (**Z**$_4$). Yellow solid; m.p. 130.5–131.8 °C; yield, 54.2%; ^{1}H-NMR (500 MHz, $CDCl_3$) δ in ppm: 8.05–8.01 (m, CO–Ph-2,6-H, Ar–CH), 7.58 (d, J = 9.16 Hz, 1H, Ar-6-H), 7.56 (d, J = 16.04 Hz, 1H, Ph–CO=CH), 6.99 (d, J = 9.16 Hz, 2H, CO–Ph-3,5-H), 6.54 (d, J = 8.59 Hz, 1H, Ar-5-H), 6.48 (s, 1H, Ar-3-H); 4.38 (t, $J_1 = J_2$ = 6.30 Hz, 2H, $–OCH_2–$), 3.90 (s, 3H, $–OCH_3$); 3.85 (s, 3H, $–OCH_3$); 3.67 (t, $J_1 = J_2$ = 6.30 Hz, 2H, $–SCH_2–$); ^{13}C-NMR (125 MHz, $CDCl_3$) δ: 189.50, 162.98, 161.54, 160.43, 140.01, 132.40, 130.98, 130.83, 120.24, 117.33, 114.38, 105.44, 98.54, 67.92, 55.65, 55.59, 28.78.

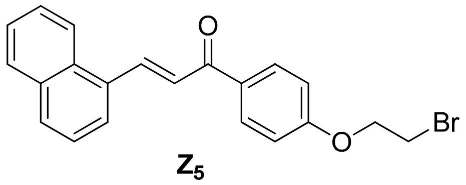

$\mathbf{Z_5}$

(E)-1-(4-(2-bromoethoxy)phenyl)-3-(naphthalen-1-yl)prop-2-en-1-one (**Z**$_5$). Yellow solid; m.p. 112.7–114.1 °C; yield, 67.8%; ^{1}H-NMR (500 MHz, $CDCl_3$) δ in ppm: 8.05 (d, J = 14.89 Hz, 1H, Ar–CH), 8.27 (d, J = 8.59 Hz, 1H, naphthalene-5-H), 8.09 (d, J = 9.16 Hz, 2H, CO–Ph-2,6-H), 7.93–7.89 (m, 3H, naphthalene-2,6,8-H), 7.64 (d, J = 15.46 Hz, 1H, Ph–CO=CH), 7.59–7.51 (m, 3H, naphthalene-3,4,7-H), 7.02 (d, J = 9.16 Hz, 2H, CO–Ph-3,5-H), 4.38 (t, $J_1 = J_2$ = 6.30 Hz, 2H, $–OCH_2–$), 3.68 (t, J_1 = 6.30 Hz, J_2 = 6.73 Hz, 2H, $–SCH_2–$); ^{13}C-NMR (125 MHz, $CDCl_3$) δ: 188.58, 161.99, 141.26, 133.83, 132.65, 131.86, 131.77, 131.06, 130.75, 128.83, 127.03, 126.39, 125.53, 125.10, 124.58, 123.66, 114.58, 67.96, 28.69.

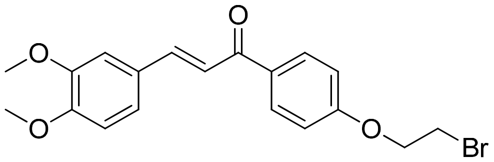

$\mathbf{Z_6}$

(E)-1-(4-(2-bromoethoxy)phenyl)-3-(3,4-dimethoxyphenyl)prop-2-en-1-one (**Z**$_6$). Yellow solid; m.p. 114.7–116.5 °C; yield, 71.2%; ^{1}H-NMR (500 MHz, $CDCl_3$) δ in ppm: 8.04 (d, J = 8.02 Hz, 2H, CO–Ph-2,6-H), 7.77 (d, J = 15.46 Hz, 1H, Ar–CH), 7.41 (d, J = 15.46 Hz, 1H, Ph–CO=CH), 7.24 (d, J = 8.02 Hz, 2H, Ar-6-H), 7.16 (s, 1H, Ar-2-H), 7.00 (d, J = 8.59 Hz, 2H, CO–Ph-3,5-H), 6.91 (d, J = 8.59 Hz, 1H, Ar-5-H), 4.37 (t, J_1 = 5.73 Hz, J_2 = 6.30 Hz, 2H, $–OCH_2–$), 3.96 (s, 3H, $–OCH_3$), 3.93 (s, 3H, $–OCH_3$), 3.67 (t, J_1 = 6.59 Hz, J_2 = 6.01 Hz, 2H, $–SCH_2–$); ^{13}C-NMR (125 MHz, $CDCl_3$) δ: 188.83, 162.77, 151.39, 149.30, 144.48, 132.00, 130.88, 128.06, 123.13, 119.78, 114.47, 111.18, 110.12, 67.94, 56.10, 56.06, 28.75.

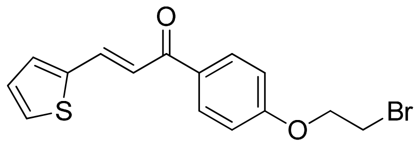

Z7

(E)-1-(4-(2-bromoethoxy)phenyl)-3-(thiophen-2-yl)prop-2-en-1-one (**Z7**). Yellow solid; m.p. 74.7–75.9 °C; yield, 65.8%; ^{1}H-NMR (500 MHz, $CDCl_3$) δ in ppm: 8.03 (d, J = 9.16 Hz, 2H, CO–Ph-2,6-H), 7.95 (d, J = 14.89 Hz, 1H, Ar–CH), 7.45 (d, J = 5.15 Hz, 1H, thiophen-5-H), 7.35 (d, J = 3.14 Hz, 1H, thiophen-3-H), 7.34 (d, J = 15.46 Hz, 1H, Ph–CO=CH), 7.08 (t, J_1 = 4.01 Hz, J_2 = 5.15 Hz, 2H, –OCH2–), 6.99 (d, J = 9.16 Hz, 2H, CO–Ph-3,5-H), 4.37 (t, J_1 = 5.73 Hz, J_2 = 6.30 Hz, 2H, –OCH_2–), 3.67 (t, J_1 = 6.01 Hz, J_2 = 6.59 Hz, 2H, –SCH_2–); ^{13}C-NMR (125 MHz, $CDCl_3$) δ: 188.13, 161.92, 140.60, 136.73, 131.99, 131.69, 130.87, 128.66, 128.42, 120.59, 114.52, 67.94, 28.73.

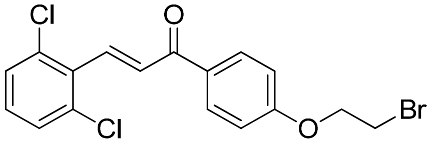

Z8

(E)-1-(4-(2-bromoethoxy)phenyl)-3-(2,6-dichlorophenyl)prop-2-en-1-one (**Z8**). Yellow solid; m.p. 127.7–129.2 °C; yield, 67.3%; ^{1}H-NMR (500 MHz, $CDCl_3$) δ in ppm: 8.05 (d, J = 8.59 Hz, 2H, CO–Ph-2,6-H), 7.86 (d, J = 16.04 Hz, 1H, Ar–CH), 7.68 (d, J = 16.04 Hz, 1H, Ph–CO=CH), 7.43 (d, J = 8.02 Hz, 2H, Ar-3,5-H), 7.21 (t, J = 8.02 Hz, 1H, Ar-4-H), 7.01 (d, J = 8.59 Hz, 2H, CO–Ph-3,5-H), 4.38 (t, J_1 = 6.01 Hz, J_2 = 6.59 Hz, 2H, –OCH_2–), 3.68 (t, J_1 = 6.30 Hz, J_2 = 5.73 Hz, 2H, –SCH2–); ^{13}C-NMR (125 MHz, $CDCl_3$) δ: 188.47, 162.16, 137.31, 135.25, 132.85, 131.31, 131.24, 130.40, 129.84, 128.93, 114.62, 67.96, 28.68.

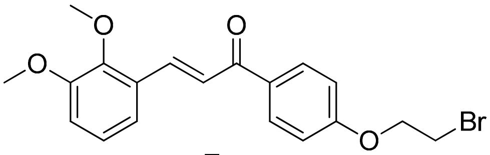

Z9

(E)-1-(4-(2-bromoethoxy)phenyl)-3-(2,3-dimethoxyphenyl)prop-2-en-1-one (**Z9**). Yellow solid; m.p. 104.7–106.1 °C; yield, 62.6%; ^{1}H-NMR (500 MHz, $CDCl_3$) δ in ppm: 8.10 (d, J = 15.46 Hz, 1H, Ar–CH), 8.05 (d, J = 9.16 Hz, 2H, CO–Ph-2,6-H), 7.62 (d, J = 15.46 Hz, 1H, Ph–CO=CH), 7.28 (d, J = 8.02 Hz, 1H, Ar-6-H), 7.10 (t, J_1 = J_2 = 8.02 Hz, 1H, Ar-5-H), 7.00 (d, J = 8.59 Hz, 2H, CO–Ph-3,5-H), 6.98 (d, J = 8.59 Hz, 1H, Ar-4-H), 4.37 (t, J_1 = 6.30 Hz, J_2 = 5.73 Hz, 2H, –OCH_2–), 3.89 (s, 3H, –OCH_3), 3.88 (s, 3H, –OCH_3), 3.67 (t, J_1 = J_2 = 6.30 Hz, 2H, –SCH_2–); ^{13}C-NMR (125 MHz, $CDCl_3$) δ: 189.15, 161.86, 153.32, 148.95, 139.13, 131.89, 131.00, 129.33, 124.30, 123.47, 119.68, 114.48, 114.07, 67.93, 61.44, 55.98, 28.67.

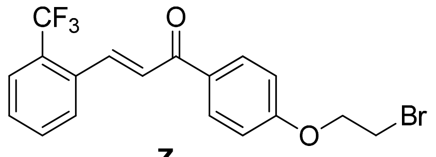

Z10

(E)-1-(4-(2-bromoethoxy)phenyl)-3-(2-(trifluoromethyl)phenyl)prop-2-en-1-one (**Z10**). Yellow solid; m.p. 144.3–146.1 °C; yield, 61.2%; ^{1}H-NMR (500 MHz, $CDCl_3$) δ in ppm: 8.13 (d, J = 15.46 Hz, 1H, Ar–CH), 8.03 (d, J = 9.16 Hz, 2H, CO–Ph-2,6-H), 7.83 (d, J = 8.02 Hz, 1H, Ar-3-H), 7.73 (d, J = 7.45 Hz, 1H, Ar-6-H), 7.60 (t, J_1 = 8.02 Hz, J_2 = 7.45 Hz, 1H, Ar-5-H), 7.50 (t, J_1 = 8.02 Hz, J_2 = 7.45 Hz, 1H, Ar-4-H), 7.43 (d, J = 15.46 Hz, 1H, Ph–CO=CH), 7.01 (d, J = 9.16 Hz, 2H, CO–Ph-3,5-H), 4.38 (t, J_1 = J_2 = 6.30 Hz, 2H, –OCH_2–), 3.67 (t, J_1 = 6.30 Hz, J_2 = 5.73 Hz, 2H, –SCH_2–); ^{13}C-NMR (125 MHz, $CDCl_3$) δ: 188.59, 162.12, 139.64, 132.17, 131.25, 131.19, 129.65, 128.04, 126.47, 126.36, 126.32, 125.15, 122.96, 114.59, 67.96, 28.69.

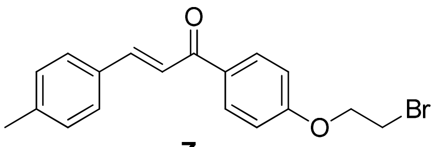

Z11

(E)-1-(4-(2-bromoethoxy)phenyl)-3-(p-tolyl)prop-2-en-1-one (**Z11**) Yellow solid; m.p. 135.2–136.9 °C; yield, 67.3%; ^{1}H-NMR (500 MHz, $CDCl_3$) δ in ppm: 8.04 (d, J = 9.16 Hz, 2H, CO–Ph-2,6-H), 7.80 (d, J = 16.04 Hz, 1H, Ar–CH), 7.55 (d, J = 8.02 Hz, 2H, Ar-2,6-H), 7.23 (d, J = 8.02 Hz, 2H, Ar-3,5-H), 7.51 (d, J = 15.46 Hz, 1H, Ph–CO=CH), 6.99 (d, J = 8.59 Hz, 2H, CO–Ph-3,5-H), 4.36 (t, J_1 = J_2 = 6.30 Hz, 2H, –OCH_2–), 3.67 (t, J_1 = 6.30 Hz, J_2 = 5.73 Hz, 2H, –SCH_2–), 2.39 (s, 3H, –CH3); ^{13}C-NMR (125 MHz, $CDCl_3$) δ: 188.86, 161.85, 144.39, 141.03, 132.34, 131.91, 130.93, 129.78, 128.52, 120.82, 114.48, 67.94, 28.77, 21.65.

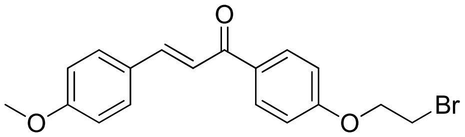

Z12

(E)-1-(4-(2-bromoethoxy)phenyl)-3-(4-methoxyphenyl)prop-2-en-1-one (**Z12**) Yellow solid; m.p. 97.6–99.2 °C; yield, 71.3%; ^{1}H-NMR (500 MHz, $CDCl_3$) δ in ppm: 8.04 (d, J = 9.16 Hz, 2H, CO–Ph-2,6-H), 7.80 (d, J = 15.46 Hz, 1H, Ar–CH), 7.61 (d, J = 8.59 Hz, 2H, Ar-2,6-H), 7.43 (d, J = 15.46 Hz, 1H, Ph–CO=CH), 6.99 (d, J = 9.16 Hz, 2H, CO–Ph-3,5-H), 6.94 (d, J = 8.59 Hz, 2H, Ar-3,5-H), 4.37 (t, J_1 = 6.30 Hz, J_2 = 5.73 Hz, 2H, –OCH_2–), 3.85 (s, 3H, –OCH_3), 3.67 (t, J_1 = J_2 = 6.01 Hz, 2H, –SCH_2–); ^{13}C-NMR (125 MHz, $CDCl_3$) δ: 186.86, 159.80, 159.69, 142.20, 130.09, 128.90, 128.30, 125.86, 117.54, 112.53, 112.51, 65.97, 53.56, 26.83.

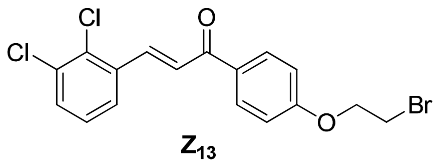

$\mathbf{Z_{13}}$

(E)-1-(4-(2-bromoethoxy)phenyl)-3-(2,3-dichlorophenyl)prop-2-en-1-one ($\mathbf{Z_{13}}$). Yellow solid; m.p. 124.7–126.2 °C; yield, 67.8%; ^{1}H-NMR (500 MHz, $CDCl_3$) δ in ppm: 8.15 (d, *J* = 15.46 Hz, 1H, Ar–CH), 8.03 (d, *J* = 8.59 Hz, 2H, CO–Ph-2,6-H), 7.64 (d, *J* = 7.45 Hz, 1H, Ar-4-H), 7.50 (d, *J* = 8.02 Hz, 1H, Ar-6-H), 7.47 (d, *J* = 16.04 Hz, 1H, Ph–CO=CH), 7.27–7.24 (m, 1H, Ar-5-H), 7.00 (d, *J* = 8.59 Hz, 2H, CO–Ph-3,5-H), 4.37 (t, J_1 = 6.30 Hz, J_2 = 5.73 Hz, 2H, –OCH_2–), 3.67 (t, J_1 = J_2 = 6.30 Hz, 2H, –SCH_2–); ^{13}C-NMR (125 MHz, $CDCl_3$) δ: 188.36, 162.16, 139.96, 135.88, 134.15, 131.58, 131.28, 131.15, 127.46, 125.98, 125.77, 116.46, 114.61, 67.97, 28.70.

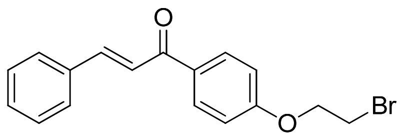

$\mathbf{Z_{14}}$

(E)-1-(4-(2-bromoethoxy)phenyl)-3-phenylprop-2-en-1-one ($\mathbf{Z_{14}}$) Yellow solid; m.p. 92.5–93.8 °C; yield, 78.8%;^{1}H-NMR (500 MHz, $CDCl_3$) δ in ppm: 8.05 (d, *J* = 9.16 Hz, 2H, CO–Ph-2,6-H), 7.82 (d, *J* = 15.46 Hz, 1H, Ar–CH), 7.66–7.62 (m, 2H, Ar-2,6-H), 7.55 (d, *J* = 15.46 Hz, 1H, Ph–CO=CH), 7.44–7.40 (m, 3H, Ar-3,4,5-H), 7.00 (d, *J* = 8.59 Hz, 2H, CO–Ph-3,5-H), 4.36 (t, J_1 = J_2 = 6.30 Hz, 2H, –OCH_2–), 3.67 (t, J_1 = 6.01 Hz, J_2 = 6.59 Hz, 2H, –SCH_2–); ^{13}C-NMR (125 MHz, $CDCl_3$) δ: 188.77, 161.93, 144.30, 135.09, 131.79, 130.97, 130.88, 129.04, 128.48, 121.87, 114.53, 67.95, 28.72.

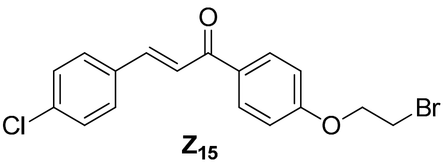

$\mathbf{Z_{15}}$

(E)-1-(4-(2-bromoethoxy)phenyl)-3-(4-chlorophenyl)prop-2-en-1-one ($\mathbf{Z_{15}}$). Yellow solid; m.p. 114.7–116.5 °C; yield, 53.2%; ^{1}H-NMR (500 MHz, $CDCl_3$) δ in ppm: 8.03 (d, *J* = 8.59 Hz, 2H, CO–Ph-2,6-H), 7.76 (d, *J* = 15.46 Hz, 1H, Ar–CH), 7.58 (d, *J* = 8.59 Hz, 1H, Ar-2,6-H), 7.52 (d, *J* = 16.04 Hz, 1H, Ph–CO=CH), 7.39 (d, *J* = 8.59 Hz, 2H, Ar-3,5-H), 7.00 (d, *J* = 8.59 Hz, 2H, CO–Ph-3,5-H), 4.37 (t, J_1 = 6.30Hz, J_2 = 6.73 Hz, 2H, –OCH_2–), 3.67 (t, J_1 = J_2 = 6.30 Hz, 2H, –SCH_2–); ^{13}C-NMR (125 MHz, $CDCl_3$) δ: 188.43, 162.03, 136.35, 133.59, 131.60, 130.98, 129.62, 128.48, 122.25, 114.57, 67.96, 28.69.

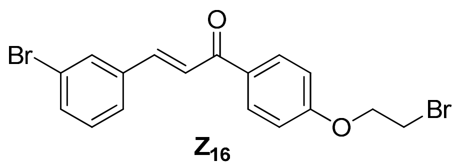

$\mathbf{Z_{16}}$

(E)-1-(4-(2-bromoethoxy)phenyl)-3-(3-bromophenyl)prop-2-en-1-one ($\mathbf{Z_{16}}$). Yellow solid; m.p. 121.1–122.8 °C; yield, 58.2%; ^{1}H-NMR (500 MHz, $CDCl_3$) δ in ppm: 8.05 (d, *J* = 7.45 Hz, 2H, CO–Ph-2,6-H), 7.79 (s, 1H, Ar-2-H), 7.73 (d, *J* = 15.46 Hz, 1H, Ar–CH), 7.29 (t, J_1 = 6.87Hz, J_2 = 7.45 Hz, 1H, Ar-5-H),

7.55–7.51 (m, 3H, Ph–CO=CH, Ar-4,6-H), 7.01 (d, J = 7.45 Hz, 2H, CO–Ph-3,5-H), 4.38 (t, J_1 = J_2 = 5.44 Hz, 2H, –OCH$_2$–), 3.67 (t, J_1 = J_2 = 6.30 Hz, 2H, –SCH$_2$–); ^{13}C-NMR (125 MHz, CDCl$_3$) δ: 188.23, 162.11, 142.45, 137.21, 133.22, 131.46, 131.05, 130.80, 130.55, 127.36, 123.16, 123.02, 114.59, 67.96, 28.71.

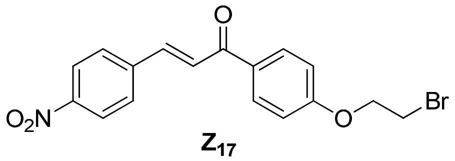

Z$_{17}$

(E)-1-(4-(2-bromoethoxy)phenyl)-3-(4-nitrophenyl)prop-2-en-1-one (**Z$_{17}$**). Yellow solid; m.p. 151.2–152.7 °C; yield, 62.8%; ^{1}H-NMR (500 MHz, CDCl$_3$) δ in ppm: 8.28 (d, J = 9.16 Hz, 2H, Ar-3,5-H), 8.06 (d, J = 8.59 Hz, 2H, CO–Ph-2,6-H), 7.82–7.77 (m, 3H, Ar–CH, Ar-2,6-H), 7.66 (d, J = 16.04 Hz, 1H, Ph–CO=CH), 7.03 (d, J = 9.16 Hz, 2H, CO–Ph-3,5-H), 4.38 (t, J_1 = 6.30 Hz, J_2 = 5.73 Hz, 2H, –OCH$_2$–), 3.67 (t, J_1 = J_2 = 6.30 Hz, 2H, –SCH$_2$–); ^{13}C-NMR (125 MHz, CDCl$_3$) δ: 187.83, 162.37, 148.54, 141.29, 141.02, 131.15, 128.97, 125.58, 124.31, 114.70, 68.00, 28.68.

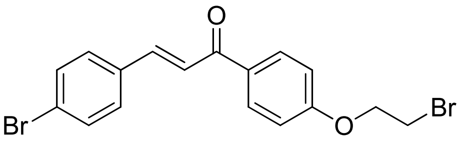

Z$_{18}$

(E)-1-(4-(2-bromoethoxy)phenyl)-3-(4-bromophenyl)prop-2-en-1-one (**Z$_{18}$**). Yellow solid; m.p. 149.1–150.5 °C; yield, 61.3%; ^{1}H-NMR (500 MHz, CDCl$_3$) δ in ppm: 8.04 (d, J = 8.59 Hz, 2H, CO–Ph-2,6-H), 7.74 (d, J = 15.46 Hz, 1H, Ar–CH), 7.56–7.49 (m, 5H, Ar-2,6-H,Ph–CO=CH, Ar-3,5-H), 7.00 (d, J = 9.16 Hz, 2H, CO–Ph-3,5-H), 4.38 (t, J_1 = 6.30Hz, J_2 = 5.73 Hz, 2H, –OCH$_2$–), 3.68 (t, J_1 = J_2 = 6.30 Hz, 2H, –SCH$_2$–); ^{13}C-NMR (125 MHz, CDCl$_3$) δ: 188.43, 162.04, 142.84, 132.27, 131.58, 130.99, 129.84, 124.73, 122.32, 114.57, 67.95, 28.70.

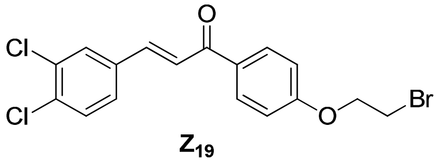

Z$_{19}$

(E)-1-(4-(2-bromoethoxy)phenyl)-3-(3,4-dichlorophenyl)prop-2-en-1-one (**Z$_{19}$**). Yellow solid; m.p. 144.2–145.5 °C; yield, 52.8%; ^{1}H-NMR (500 MHz, CDCl$_3$) δ in ppm: 8.05 (d, J = 8.59 Hz, 2H, CO–Ph-2,6-H), 7.73 (d, J = 8.59 Hz, 1H, Ar-6-H), 7.70 (d, J = 15.46 Hz, 1H, Ar–CH), 7.53 (d, J = 16.04 Hz, 1H, Ph–CO=CH), 7.48 (s, 1H, Ar-2-H), 7.46 (d, J = 8.02 Hz, 1H, Ar-5-H), 7.01 (d, J = 8.59 Hz, 2H, CO–Ph-3,5-H), 4.38 (t, J_1 = 5.73 Hz, J_2 = 6.30 Hz, 2H, –OCH$_2$–), 3.68 (t, J_1 = 6.30 Hz, J_2 = 5.73 Hz, 2H, –SCH$_2$–); ^{13}C-NMR (125 MHz, CDCl$_3$) δ: 188.04, 162.17, 141.37, 134.31, 133.36, 131.48, 131.36, 131.23, 131.05, 127.61, 123.31, 114.62, 67.97, 28.68.

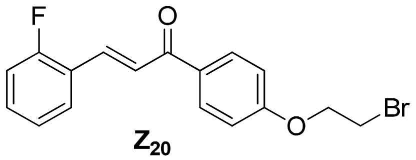

Z_{20}

(E)-1-(4-(2-bromoethoxy)phenyl)-3-(2-fluorophenyl)prop-2-en-1-one (**Z_{20}**). Yellow solid; m.p. 114.7–116.5 °C; yield, 63.8%; ^{1}H-NMR (500 MHz, $CDCl_3$) δ in ppm: 8.05 (d, J = 9.16 Hz, 2H, CO–Ph-2,6-H), 7.90 (d, J = 16.04 Hz, 1H, Ar–CH), 7.66–7.63 (m, 2H, Ph–CO=CH, Ar-4-H), 7.38 (d, J = 8.02 Hz, 1H, Ar-6-H), 7.19 (t, J_1 = 7.45 Hz, J_2 = 8.02 Hz, 1H, Ar-3-H), 7.13 (t, J_1 = 10.88 Hz, J_2 = 8.02 Hz, 1H, Ar-5-H), 7.00 (d, J = 8.59 Hz, 2H, CO–Ph-3,5-H), 4.37 (t, J_1 = J_2 = 6.30 Hz, 2H, –OCH_2–), 3.67 (t, J_1 = 6.59 Hz, J_2 = 6.01 Hz, 2H, –SCH_2–); ^{13}C-NMR (125 MHz, $CDCl_3$) δ: 188.73, 162.11, 136.61, 133.97, 131.45, 131.60, 130.75, 128.61, 122.49, 115.05, 114.86, 114.58, 67.95, 28.67.

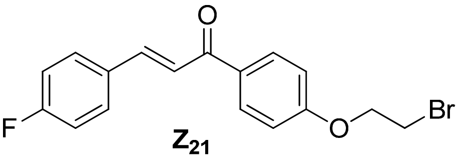

Z_{21}

(E)-1-(4-(2-bromoethoxy)phenyl)-3-(4-fluorophenyl)prop-2-en-1-one (**Z_{21}**). Yellow solid; m.p. 113.3–114.8 °C; yield, 62.5%; ^{1}H-NMR (500 MHz, $CDCl_3$) δ in ppm: 8.04 (d, J = 8.59 Hz, 2H, CO–Ph-2,6-H), 7.78 (d, J = 15.46 Hz, 1H, Ar–CH), 7.62 (t, J_1 = 8.59 Hz, J_2 = 5.15 Hz, 2H, Ar-2,6-H), 7.47 (d, J = 15.46 Hz, 1H, Ph–CO=CH), 7.10 (t, J_1 = J_2 = 8.59 Hz, 2H, Ar-3,5-H), 7.00 (d, J = 8.59 Hz, 2H, CO–Ph-3,5-H), 4.37 (t, J_1 = 6.30 Hz, J_2 = 5.73 Hz, 2H, –OCH_2–), 3.67 (t, J_1 = 6.30 Hz, J_2 = 5.73 Hz, 2H, –SCH_2–); ^{13}C-NMR (125 MHz, $CDCl_3$) δ: 188.51, 161.96, 142.95, 131.70, 130.95, 130.38, 130.32, 121.54, 116.28, 116.10, 114.55, 67.96, 28.70.

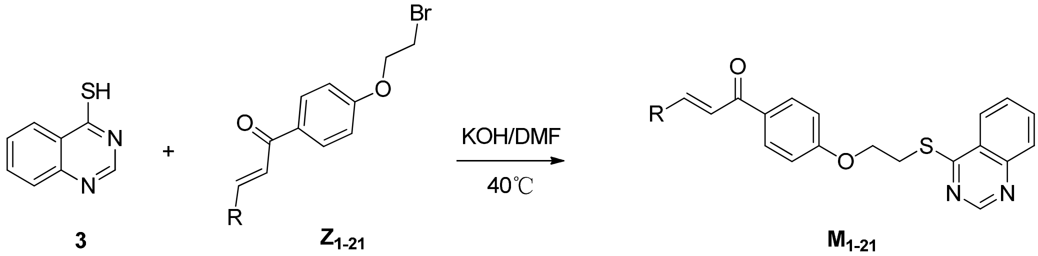

3 Z_{1-21} M_{1-21}

General Procedure for Preparation of Title Compounds (M_1–M_{21})

The target compounds **M_1–M_{21}** were synthesized as schematized in Figure 2. A 25 mL round-bottomed flask equipped with a magnetic stirrer was charged with intermediates **3** (0.74 mmol) and KOH (0.89 mmol), DMF (5 mL). The flask was stirred at 40 °C for 1 h, and then **Z** (0.59 mmol) in DMF (2 mL) was dropwise into the flask. The resulting mixture was stirred at 40 °C for 8 to 10 h. TLC monitored the progress of the reaction. Upon completion of the reaction (as indicated by TLC), the reaction mixture was poured into saturated brine, the solid was filtered off. Then dissolved with dichloromethane and washed by 10% KOH. The organic fraction was evaporated under reduced pressure. The solid was recrystallized from ethyl acetate/petroleum ether (3:1, *v*/*v*) to obtain the title compounds **M_1–M_{21}** in 56.1% to 78.8% yields.

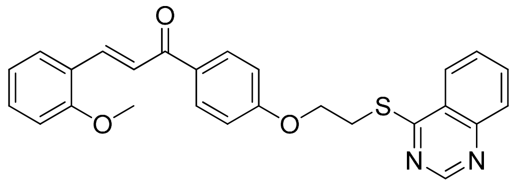

M1

(E)-3-(2-methoxyphenyl)-1-(4-(2-(quinazolin-4-ylthio)ethoxy)phenyl)prop-2-en-1-one (**M1**). Yellow solid; m.p. 114.7–116.5 °C; yield, 78.8%; IR (KBr, cm^{-1}) *v*: 3016.8–3066.9 (C–H of benzene), 1661.7 (C=N), 1604.8 (C=O), 1485.2–1560.4 (C=C and benzene and Qu-ring), 1319.3 (C–N), 1249.9 (C–O), 1150.5 (–O–CH3); ^{1}H-NMR (500 MHz, $CDCl_3$) δ in ppm: 9.02 (s, 1H, Qu-2-H), 8.12–8.08 (m, 2H, CH=C-Ph, Qu-8-H), 8.05 (d, J = 8.60 Hz, 2H, Ph-2-CH_3-6-H), 7.99 (d, J = 8.60 Hz, 1H, Qu-8-H), 7.87 (t, J_1 = 8.60 Hz, J_2 = 6.85 Hz, 1H, Qu-7-H), 7.64–7.59 (m, 3H, Qu-6,7-H,Ph–CO–CH), 7.37 (t, J_1 = 6.9 Hz, J_2 = 7.45 Hz, 1H, Ph-2-OCH_3-4-H), 7.07 (d, J = 9.15 Hz, 2H, Ph-2-OCH_3-2,6-H), 6.99 (t, J = 7.45 Hz, 1H, Ph-2-OCH_3-5-H), 6.95 (d, J = 8.55 Hz, 1H, Ph-2-OCH_3-3-H), 4.42 (t, J_1 = 6.60 Hz, J_2 = 6.55 Hz, 2H, –OCH_2–), 3.91 (s, 3H,–CH_3), 3.82 (t, J_1 = 6.85 Hz, J_2 = 6.30 Hz, 2H, –SCH_2–); ^{13}C-NMR (125 MHz, $CDCl_3$) δ in ppm:189.42, 170.40, 162.17, 158.12, 153.51, 148.17, 139.75, 134.04, 131.80, 131.65, 130.95, 129.26, 128.98, 127.64, 124.16, 123.99, 122.73, 120.80, 114.46, 111.29, 66.53, 55.63, 28.33; Anal. Calcd for $C_{26}H_{22}N_2O_3S$: C, 68.62; H, 5.01; N, 6.33; Found: C, 70.12; H, 5.53; N, 6.44.

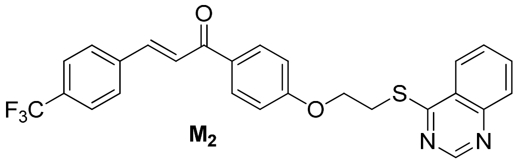

M2

(E)-1-(4-(2-(quinazolin-4-ylthio)ethoxy)phenyl)-3-(4-(trifluoromethyl)phenyl)prop-2-en-1-one (**M2**). Yellow solid; m.p. 187.9–188.8 °C; yield, 71.9%; IR (KBr, cm^{-1}) *v*: 3046.7–3071.7 (C–H of benzene), 1663.6 (C=N), 1610.6 (C=O), 1486.2–1562.4 (C=C and benzene and Qu-ring), 1328.0 (C–N), 1255.7 (C–O); ^{1}H-NMR (500 MHz, $CDCl_3$) δ in ppm: 9.02 (s, 1H, Qu-2-H), 8.10 (d, 1H, J = 8.00 Hz, Qu-8-H), 8.06 (d, J = 9.15 Hz, 2H, CO–Ph-2,6-H), 7.99 (d, J = 8.60 Hz, 1H, Qu-5-H), 7.87 (t, J_1 = 8.60 Hz, J_2 = 6.85 Hz, 1H, Qu-7-H), 7.81 (d, J = 16.05 Hz, 1H, Ar–CH), 7.74 (d, J = 8.00 Hz, 2H, Ar-2,6-H), 7.67 (d, J = 8.60Hz, 2H, Ar-2,6-H), 7.62–7.58 (m, Ph–CO=CH, Qu-6-H), 7.09 (d, 2H, J = 8.60 Hz, CO–Ph-3,5-H), 4.43 (t, 2H, J_1 = 6.30 Hz, J_2 = 6.85Hz, –OCH_2–), 3.82 (t, 2H, J_1 = 6.90 Hz, J_2 = 6.30 Hz, –SCH_2–); ^{13}C-NMR (125 MHz, $CDCl_3$) δ in ppm:188.21, 170.31, 162.64, 153.49, 148.20, 142.08, 138.54, 134.04, 131.11, 131.04, 130.97, 129.01, 128.50, 127.66, 125.97, 125.94, 124.13, 123.98, 123.85, 114.67, 66.63, 28.27; Anal. Calcd for $C_{26}H_{19}N_2O_2S$: C, 64.99; H, 3.99; N, 5.83; Found: C, 64.56; H, 4.25; N, 6.09.

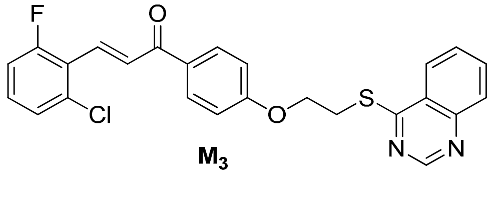

M3

(E)-3-(2-chloro-6-fluorophenyl)-1-(4-(2-(quinazolin-4-ylthio)ethoxy)phenyl)prop-2-en-1-one (**M3**). Yellow solid; m.p. 109.5–110.8 °C; yield, 61.2%; IR (KBr, cm^{-1}) *v*: 3041.8–3082.3 (C–H of benzene), 1666.5

(C=N), 1609.6 (C=O), 1486.2–1560.4 (C=C and benzene and Qu-ring), 1319.3 (C–N), 1257.6 (C–O); ^{1}H-NMR (500 MHz, $CDCl_3$) δ in ppm:9.02 (s, 1H, Qu-2-H), 8.11 (d, J = 8.59 Hz, 1H, Qu-8-H), 8.05 (d, J = 9.16 Hz, 2H, CO–Ph-2,6-H), 7.99 (d, J = 8.02 Hz, 1H, Qu-5-H), 7.97 (d, J = 16.04 Hz, 1H, Ar–CH), 7.87 (t, J_1 = 8.02 Hz, J_2 = 7.45 Hz, 1H, Qu-7-H), 7.82 (d, J = 16.04 Hz, 1H, Ph–CO=CH), 7.61 (t, J_1 = 8.02 Hz, J_2 = 7.45 Hz, 1H, Qu-6-H), 7.33–7.21 (m, 3H, Ar-3-4-5-H), 7.08 (d, J = 9.16 Hz, 2H, CO–Ph-3,5-H), 4.43 (t, J_1 = 6.87 Hz, J_2 = 6.30 Hz, 2H, $–OCH_2–$), 3.82 (t, J_1 = J_2 = 6.30 Hz, 2H, $–SCH_2–$); ^{13}C-NMR (125 MHz, $CDCl_3$) δ in ppm:188.74, 170.36, 162.56, 153.08, 148.18, 136.63, 134.03, 133.85, 131.14, 130.70, 130.62, 128.98, 128.68, 128.57, 127.65, 126.19, 123.99, 123.88, 115.04, 114.84, 114.62, 66.59, 28.30; Anal. Calcd for $C_{25}H_{18}ClFN_2O_2S$: C, 64.58; H, 3.90; N, 6.03; Found: C, 65.04; H, 3.32; N, 5.62.

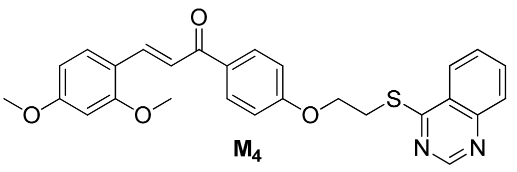

$\mathbf{M_4}$

(E)-3-(2,4-dimethoxyphenyl)-1-(4-(2-(quinazolin-4-ylthio)ethoxy)phenyl)prop-2-en-1-one (**M**$_4$). Yellow solid; m.p. 103.5–104.9 °C; yield, 56.7%; IR (KBr, cm^{-1}) v: 3010.0–3040.9 (C–H of benzene), 1648.2 (C=N), 1602.9 (C=O), 1458.2–1565.3 (C=C and benzene and Qu-ring), 1323.2 (C–N), 1250.8 (C–O), 1183.3 (–O–CH3); ^{1}H-NMR (500 MHz, $CDCl_3$) δ: 9.02 (s, 1H, Qu-2-H), 8.11 (d, J = 8.02 Hz, 1H,Qu-8-H), 8.05–8.02 (m, 3H, CO–Ph-2,6-H, Ar–CH), 7.99 (d, J = 8.02 Hz, 1H,Qu-5-H), 7.87 (t, J_1 = J_2 = 6.87 Hz, 1H, Qu-7-H), 7.61 (t, J_1 = 8.02 Hz, J_2 = 7.45 Hz, 1H, Qu-6-H), 7.58–7.53 (m, 2H, Ar-6-H, Ph–CO=CH), 7.06 (d, J = 8.59 Hz, 2H, CO–Ph-3,5-H), 6.54 (d, J = 8.02 Hz, 1H, Ar-5-H), 6.54 (s, 1H, Ar-3-H), 4.42 (t, J_1 = J_2 = 6.59 Hz, 2H, $–OCH_2–$), 3.90 (s, 3H, $–CH_3$), 3.85 (s, 3H, $–CH_3$), 3.82 (t, J_1 = 6.01 Hz, J_2 = 7.16 Hz, 2H, $–SCH_2–$); ^{13}C-NMR (125 MHz, $CDCl_3$) δ: 189.44, 170.34, 162.86, 161.91, 160.32, 153.43, 148.09, 139.80, 133.93, 132.03, 130.86, 130.74, 128.89, 127.55, 123.93, 123.81, 120.23, 117.29, 114.32, 105.34, 98.46, 66.44, 55.56, 55.50, 28.28; Anal. Calcd for $C_{27}H_{24}N_2O_4S$: C, 68.62; H, 5.12; N, 5.93; Found: C, 68.40; H, 5.04; N, 5.86.

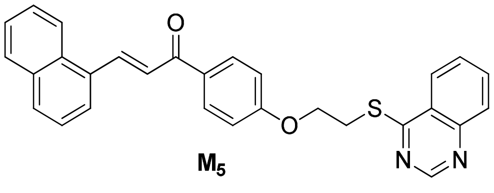

$\mathbf{M_5}$

(E)-3-(naphthalen-1-yl)-1-(4-(2-(quinazolin-4-ylthio)ethoxy)phenyl)prop-2-en-1-one (**M**$_5$). Yellow solid; m.p. 168.5–170.1 °C; yield, 67.6%; IR (KBr, cm^{-1}) v: 3059.2–3040.9 (C–H of benzene), 1651.1 (C=N), 1604.8 (C=O), 1486.2–1561.4 (C=C and benzene and Qu-ring), 1349.2 (C–N), 1256.6 (C–O); ^{1}H-NMR (500 MHz, $CDCl_3$) δ in ppm: 9.02 (s, 1H, Qu-2-H), 8.66 (d, J = 15.45 Hz, 1H, Ar–CH), 8.27 (d, J = 8.60 Hz, 2H, Qu-8-H), 8.10–8.08 (m, 3H, CO–Ph-2,6-H, Ar-5), 7.98 (d, J = 8.00 Hz, 1H, Ar-4-H), 7.92–7.85 (m, 4H, Ar-5,8-H, Qu-5,7-H), 7.64–7.50 (m, 5H, Ph–CO=CH, Ar-3,6,7-H, Qu-6-H), 7.09 (d, J = 9.15 Hz, 2H, CO–Ph-3,5-H), 4.43 (t, J_1 = J_2 = 6.30 Hz, 2H, $–OCH_2–$), 3.82 (t, J_1 = 6.85 Hz, J_2 = 6.30 Hz, 2H, $–SCH_2–$); ^{13}C-NMR (125 MHz, $CDCl_3$) δ in ppm:188.47, 170.25, 162.35, 153.42, 148.11, 141.03, 133.93, 133.72, 132.59, 131.76, 131.38, 130.95, 128.91, 128.72, 127.55, 126.91,

126.27, 125.43, 124.99, 124.54, 123.91, 123.78, 123.78, 123.57, 114.52, 66.50, 28.22; Anal. Calcd for $C_{29}H_{22}N_2O_2$ S: C, 75.30; H, 4.79; N, 6.06; Found: C, 75.12; H, 4.64; N, 5.93.

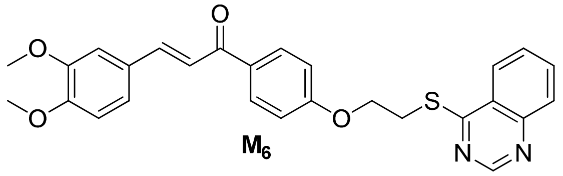

M6

(E)-3-(3,4-dimethoxyphenyl)-1-(4-(2-(quinazolin-4-ylthio)ethoxy)phenyl)prop-2-en-1-one (**M**$_6$). Yellow solid; m.p. 151.2–153.1 °C; yield, 72.5%; IR (KBr, cm^{-1}) *v*: 3023.5–3068.8 (C-H of benzene), 1654.0 (C=N), 1604.9 (C=O), 1490.0–1562.4 (C=C and benzene and Qu-ring), 1328.0 (C-N), 1260.5 (C–O); 1172.7 (–O–CH_3); ^{1}H-NMR (500 MHz, $CDCl_3$) δ: 9.01 (s, 1H, Qu-2-H), 8.10 (d, *J* = 7.45 Hz, 1H,Qu-8-H), 8.04 (d, *J* = 9.15 Hz, 2H, CO–Ph-2,6-H), 7.98 (d, *J* = 8.55 Hz, 1H, Qu-5-H), 7.86 (t, J_1 = 8.60 Hz, J_2 = 6.85 Hz, 1H, Qu-7-H), 7.77 (d, 1H, *J* = 15.45 Hz, Ar–CH), 7.60 (t, J_1 = 7.40 Hz, J_2 = 6.90 Hz, 1H, Qu-6-H), 7.41 (d, *J* = 15.45 Hz, 1H, Ph–CO=CH), 7.23 (dd, J_1 = J_2 = 1.70 Hz, 1H, Ar-2-H), 7.15 (d, *J* = 1.75 Hz, 1H, Ar-6-H), 7.07 (d, *J* = 8.60 Hz, 2H, CO–Ph-3,5-H), 4.42 (t, J_1 = 6.60, J_2 = 7.40 Hz, 2H, –OCH_2–), 3.95 (s, 3H, –CH_3), 3.92 (s, 3H, –CH_3), 3.81 (t, J_1 = 7.35 Hz, J_2 = 6.30 Hz, 2H, –SCH_2–); ^{13}C-NMR (125 MHz, $CDCl_3$) δ: 188.71, 170.25, 162.13, 153.41, 151.26, 149.20, 148.09, 144.24, 133.92, 131.62, 130.76, 128.90, 128.01, 127.54, 123.89, 123.77, 122.98, 119.75, 114.41, 111.10, 110.07, 66.46, 55.99, 55.97, 28.22; Anal. Calcd for $C_{27}H_{24}N_2O_4S$: C, 68.62; H, 5.12; N, 5.93; Found: C, 68.30; H, 5.11; N, 5.91.

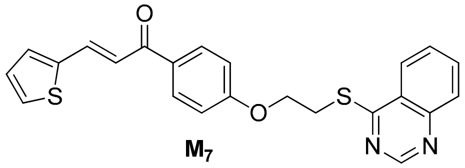

M7

(E)-1-(4-(2-(quinazolin-4-ylthio)ethoxy)phenyl)-3-(thiophen-2-yl)prop-2-en-1-one (**M**$_7$) Yellow solid; m.p. 173.1–174.6 °C; yield, 67.4%; IR (KBr, cm^{-1}) *v*: 3022.5–3076.5 (C–H of benzene), 1654.0 (C=N), 1601.9 (C=O), 1485.2–1589.4 (C=C and benzene and Qu-ring), 1321.3 (C–N), 1255.7 (C–O); ^{1}H-NMR (500 MHz, $CDCl_3$) δ in ppm: 9.01 (s, 1H, Qu-2-H), 8.09 (d, *J* = 8.00 Hz, 1H, Qu-8-H), 8.02 (d, *J* = 9.15 Hz, 2H, CO–Ph-2,6-H), 7.98 (d, *J* = 8.05 Hz, 1H, Qu-5-H), 7.94 (d, *J* = 15.45 Hz, 1H, Ar–CH), 7.86 (t, J_1 = 8.60 Hz, J_2 = 6.90 Hz, 1H, Qu-7-H), 7.59 (t, 1H, J_1 = 8.00 Hz, J_2 = 7.45 Hz, Qu-6-H), 7.40 (d, 1H, *J* = 4.60 Hz, Ar-5-H), 7.34–7.31 (m, 2H, Ar-3,4-H), 7.08–7.04 (m, 3H,Ph–CO=CH, CO–Ph-3,5-H), 4.41 (t, J_1 = 6.30 Hz J_2 = 6.85Hz, 2H, –OCH_2–), 3.81 (t, J_1 = 6.85 Hz, J_2 = 6.30 Hz, 2H, –SCH_2–); ^{13}C-NMR (125 MHz, $CDCl_3$) δ in ppm:187.99, 170.24, 162.25, 153.40, 148.08, 140.52, 136.48, 133.90, 131.78, 131.30, 130.73, 128.89, 128.48, 128.29, 127.52, 123.88, 123.76, 120.55, 114.45, 66.47, 28.21; Anal. Calcd for $C_{23}H_{18}N_2O_2$ S_2: C, 66.00; H, 4.33; N, 6.69; Found: C, 65.59; H, 4.66; N, 6.64.

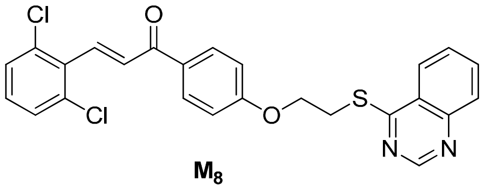

M8

(E)-3-(2,6-dichlorophenyl)-1-(4-(2-(quinazolin-4-ylthio)ethoxy)phenyl)prop-2-en-1-one (**M8**) Yellow solid; m.p. 160.5–162.3 °C; yield, 78.6%; IR (KBr, cm^{-1}) *v*: 3032.2–3081.4 (C–H of benzene), 1659.8 (C=N), 1614.4 (C=O), 1486.2–1563.3 (C=C and benzene and Qu-ring), 1333.8 (C–N), 1260.5 (C–O); ^{1}H-NMR (500 MHz, $CDCl_3$) δ in ppm: 9.01 (s, 1H, Qu-2-H), 8.10 (d, *J* = 8.05 Hz, 1H, Qu-8-H), 8.04 (d, *J* = 9.15 Hz, 2H, CO–Ph-2,6-H), 7.98 (d, *J* = 8.00 Hz, 1H, Qu-5-H), 7.88 (d, *J* = 8.59 Hz, 1H, Ar-4-H), 7.85 (d, *J* = 16.04 Hz, 1H, Ar–CH), 7.68 (d, *J* = 16.05 Hz, 1H, Ph–CO=CH), 7.60 (t, J_1 = 8.02 Hz, J_2 = 7.45 Hz, 1H, Qu-7-H), 7.38 (d, *J* = 8.00 Hz, 2H, Ar-3,5-H), 7.19 (t, J_1 = 8.02 Hz, J_2 = 8.59 Hz, 1H, Qu-6-H), 7.08 (d, *J* = 9.20 Hz, 2H, CO–Ph-3,5-H), 4.42 (t, J_1 = 6.85 Hz, J_2 = 6.30 Hz, 2H, –OCH_2–), 3.81 (t, J_1 = 6.30 Hz, J_2 = 6.85 Hz, 2H,–SCH_2–); ^{13}C-NMR (125 MHz, $CDCl_3$) δ in ppm:188.37, 170.24, 162.50, 153.40, 148.06, 137.09, 135.12, 133.93, 132.78, 131.12, 130.91,130.34, 129.71, 128.88, 128.81, 127.55, 123.87, 123.76, 114.53, 66.48, 28.18; Anal. Calcd for $C_{25}H_{18}Cl_2N_2O_4S$: C, 62.37; H, 3.77; N, 5.82; Found: C, 61.12; H, 3.72; N, 5.72.

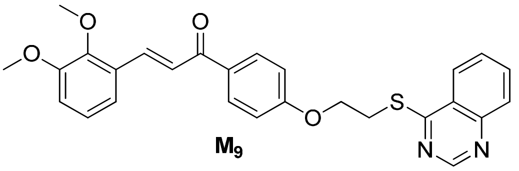

M9

(E)-3-(2,3-dimethoxyphenyl)-1-(4-(2-(quinazolin-4-ylthio)ethoxy)phenyl)prop-2-en-1-one (**M9**) Yellow solid; m.p. 174–176 °C; yield, 48.6%; IR (KBr, cm^{-1}) *v*: 3016.8-3066.9 (C–H of benzene), 1661.7 (C=N), 1608.5 (C=O), 1485.2–1560.4 (C=C and benzene and Qu-ring), 1319.3 (C–N), 1249.9 (C–O), 1150.5 (–O–CH_3); ^{1}H-NMR (500 MHz, $CDCl_3$) δ in ppm: 9.02 (s, 1H, Qu-2-H), 8.10 (d, *J* = 7.45 Hz, 1H, Qu-8-H), 8.10 (d, *J* = 16.04 Hz, 1H, Ar–CH), 8.05 (d, *J* = 9.16 Hz, 2H, CO–Ph-2,6-H), 7.99 (d, *J* = 8.59 Hz, 1H, Qu-5-H), 7.87 (t, J_1 = 6.87 Hz, J_2 = 7.45 Hz, 1H, Qu-7-H), 7.62–7.59 (m, 2H, Ph–CO=CH, Qu-6-H), 7.28 (d, *J* = 8.02 Hz, 1H, Ar-6-H), 7.10 (d, J_1 = J_2 = 8.02 Hz, 1H, Ar-5-H), 7.07 (d, *J* = 8.59 Hz, 2H, CO–Ph-3,5-H), 6.97 (d, *J* = 6.87 Hz, 1H, Ar-4-H), 4.42 (t, J_1 = 6.30 Hz, J_2 = 6.87 Hz, 2H, –OCH_2–), 3.89 (s, 3H, –OCH_3), 3.88 (s, 3H, –OCH_3), 3.82 (t, J_1 = 6.87 Hz, J_2 = 6.30 Hz, 2H, –SCH_2–); ^{13}C-NMR (125 MHz, $CDCl_3$) δ in ppm:189.14, 170.38, 162.30, 153.51, 153.31, 148.93, 148.17, 138.99, 134.04, 131.59, 130.98, 129.37, 128.98, 127.65, 124.28, 123.99, 119.67, 114.52, 114.03, 66.55, 61.44, 55.98, 28.31; Anal. Calcd for $C_{27}H_{22}N_2O_4S$: C, 68.62; H, 5.12; N, 5.93; Found: C, 68.32; H, 5.08; N, 5.78.

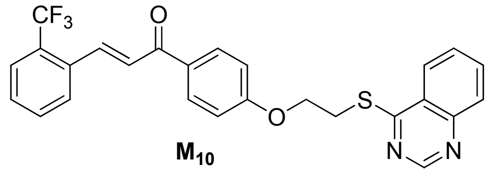

M10

(E)-1-(4-(2-(quinazolin-4-ylthio)ethoxy)phenyl)-3-(2-(trifluoromethyl)phenyl)prop-2-en-1-one (**M10**) Yellow solid; m.p. 151.4–153.2 °C; yield, 72.5%; IR (KBr, cm^{-1}) *v*: 3032.3-3072.3 (C–H of benzene), 1668.5 (C=N), 1616.4 (C=O), 1482.3–1558.5 (C=C and benzene and Qu-ring), 1319.3 (C–N), 1276.9 (C–O); ^{1}H-NMR (500 MHz, $CDCl_3$) δ in ppm: 9.02 (s, 1H, Qu-2-H), 8.12–8.09 (m, 2H, Ar–CH, Qu-8-H), 8.04 (d, *J* = 8.6 Hz, 2H, CO–Ph-2,6-H), 7.99 (d, *J* = 8.05 Hz, 1H, Qu-5-H), 7.87 (t, J_1 = 6.85 Hz, J_2 = 7.45 Hz, 1H, Qu-7-H), 7.83 (d, *J* = 7.45 Hz, 1H,Ar-3-H), 7.73 (d, *J* = 8.00 Hz, 1H, Ar-6-H), 7.62–7.58 (m, 2H, Ar-4,5-H), 7.49 (t, J_1 = 8.00 Hz, J_2 = 7.45 Hz, 1H, Qu-6-H), 7.44 (d, *J* = 15.45 Hz, 1H, Ph–CO–CH), 7.08 (d, *J* = 9.15 Hz, 2H, CO–Ph-3,5-H), 4.42 (t, J_1 = 6.30 Hz, J_2 = 6.90 Hz, 2H, –OCH_2–), 3.82 (t, J_1 = 6.85 Hz, J_2 = 6.30 Hz, 2H, –SCH_2–); ^{13}C-NMR (125 MHz, $CDCl_3$) δ in ppm:188.47, 170.25, 162.47, 153.41, 148.07, 139.43, 134.20, 133.96, 132.06, 131.08, 130.85,129.52, 129.00, 128.89, 127.94, 127.57, 126.41, 126.25, 126.21, 123.89, 123.78, 114.52, 66.49, 28.18; Anal. Calcd for $C_{26}H_{19}F_3N_2O_2S$: C, 64.99; H, 3.99; N, 5.83; Found: C, 64.46; H, 4.27; N, 5.88.

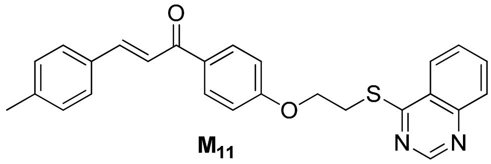

M11

(E)-1-(4-(2-(quinazolin-4-ylthio)ethoxy)phenyl)-3-(p-tolyl)prop-2-en-1-one (**M11**) Yellow solid; m.p. 153.3–154.9 °C; yield, 56.7%; IR (KBr, cm^{-1}) *v*:3023.5–3059.3 (C–H of benzene), 2943.5 (–CH_3); 1654.0 (C=N), 1601.9 (C=O), 1486.2–1565.3 (C=C and benzene and Qu-ring), 1323.2 (C–N), 1264.3 (C–O); ^{1}H-NMR (500 MHz, $CDCl_3$) δ in ppm: 9.01 (s, 1H, Qu-2-H), 8.09 (d, *J* = 8.02 Hz, 1H, Qu-8-H), 8.04 (d, *J* = 9.16 Hz, 2H, CO–Ph-2,6-H), 7.98 (d, *J* = 8.59 Hz, 1H, Qu-5-H), 7.87 (t, J_1 = J_2 = 6.87 Hz, 1H, Qu-7-H), 7.78 (d, *J* = 16.04 Hz, 1H, Ar–CH), 7.60 (t, J_1 = 6.87 Hz, J_2 = 8.02Hz, 1H, Qu-6-H), 7.55 (d, *J* = 8.02 Hz, 2H, Ar-2,6-H), 7.52 (d, *J* = 15.46 Hz, 2H, Ph–CO=CH), 7.23 (d, *J* = 8.02 Hz, 2H, Ar-3,5-H), 7.06 (d, *J* = 8.59 Hz, 2H, CO–Ph-3,5-H), 4.42 (t, J_1 = 6.30 Hz, J_2 = 6.87Hz, 2H, –OCH_2–), 3.82 (t, J_1 = 6.87 Hz, J_2 = 6.30 Hz, 2H, –SCH_2–), 2.38 (s, 3H, –CH_3); ^{13}C-NMR (125 MHz, $CDCl_3$) δ in ppm: 188.88, 170.37, 162.28, 153.52, 148.18, 144.28, 140.97, 134.04, 132.38, 131.62, 130.91, 129.76, 128.99, 128.50, 127.65, 123.99, 123.89, 120.86, 114.52, 66.55, 28.31, 21.64; Anal. Calcd for $C_{26}H_{22}N_2O_2S$: C, 73.21; H, 5.20; N, 6.57; Found: C, 73.08; H, 5.12; N, 6.26.

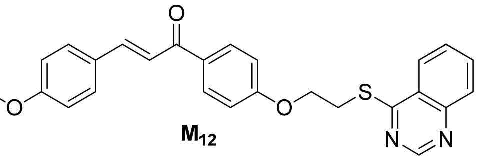

M12

(E)-3-(4-methoxyphenyl)-1-(4-(2-(quinazolin-4-ylthio)ethoxy)phenyl)prop-2-en-1-one (**M12**) Yellow solid; m.p. 109.5–111.4 °C; yield, 62.3%; IR (KBr, cm^{-1}) *v*: 3017.3–3067.5 (C–H of benzene), 2930.0–2946.3 (–O–CH_3), 1653.0 (C=N), 1602.9 (C=O), 1490.7–1563.3 (C=C and benzene and Qu-ring), 1326.1 (C–N),

1264.3 (C–O),1168.9 (–CH_3); ^{1}H-NMR (500 MHz, $CDCl_3$) δ in ppm: 9.02 (s, 1H, Qu-2-H), 8.10 (d, J = 8.02 Hz, 1H, Qu-8-H), 8.04 (d, J = 8.59 Hz, 2H, CO–Ph-2,6-H), 7.99 (d, J = 8.59 Hz, 1H, Qu-5-H), 7.87 (t, J_1 = 8.59 Hz, J_2 = 6.87 Hz, 1H, Qu-7-H), 7.79 (d, J = 15.46 Hz, 1H, Ar–CH), 7.62–7.59 (m, 3H, Qu-6-H, Ar-2,6-H), 7.44 (d, J = 15.46 Hz, 1H, Ph–CO=CH), 7.07 (d, J = 9.16 Hz, 2H, CO–Ph-3,5-H), 6.94 (d, J = 9.16 Hz, 2H, Ar-3,5-H), 4.42 (t, J_1 = J_2 = 6.59 Hz, 2H, –OCH_2–), 3.85 (s, 3H, –OCH_3), 3.82 (t, J_1 = J_2 = 6.30 Hz, 2H, –SCH_2–);^{13}C-NMR (125 MHz, $CDCl_3$) δ in ppm:188.81, 170.38, 162.19, 161.60, 153.51, 148.17, 144.03, 134.04, 131.75, 130.84, 130.23, 128.99,127.84, 127.65, 127.56, 123.89, 119.54, 114.48, 114.46, 66.53, 55.51, 28.31; Anal. Calcd for $C_{26}H_{22}N_2O_3S$: C, 70.57; H, 5.01; N, 6.33; Found: C, 70.91; H, 4.73; N, 5.96.

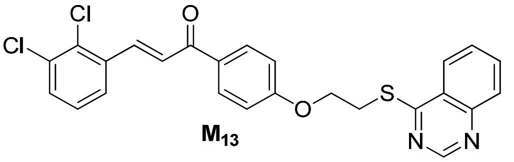

M13

(E)-3-(2,3-dichlorophenyl)-1-(4-(2-(quinazolin-4-ylthio)ethoxy)phenyl)prop-2-en-1-one (**M13**) Yellow solid; m.p. 141.3–123.1 °C; yield, 71.1%; IR (KBr, cm^{-1}) v: 3040.9–3058.2 (C–H of benzene), 1662.7 (C=N), 1606.7 (C=O), 1419.6–1560.4 (C=C and benzene and Qu-ring), 1330.9 (C–N), 1250.8 (C–O); ^{1}H-NMR (500 MHz, $CDCl_3$) δ in ppm: 9.02 (s, 1H, Qu-2-H), 8.16 (d, 1H, J = 15.45 Hz, Ar–CH), 8.11 (d, 1H, J = 7.45 Hz, Qu-8-H), 8.04 (d, 2H, J = 8.60 Hz, CO–Ph-2,6-H), 7.99 (d, 1H, J = 8.05 Hz, Qu-5-H), 7.88 (t, 1H, J_1 = 6.85 Hz, J_2 = 8.60 Hz, Qu-7-H), 7.65–7.61 (m, 2H, Ar-4,6-H), 7.51 (t, 1H, J_1 = 8.00 Hz, J_2 = 7.40 Hz, Qu-6-H), 7.26–7.22 (m, 1H, Ar-5-H), 7.47 (d, 1H, J = 15.45 Hz, Ph–CO–CH), 7.08 (d, 2H, J = 8.60 Hz, CO–Ph-3,5-H,), 4.43 (t, 2H, J_1 = 6.30 Hz, J_2 = 6.85 Hz, –OCH_2–), 3.82 (t, 2H, J_1 = 6.85 Hz, J_2 = 6.30 Hz, –SCH_2–); ^{13}C-NMR (125 MHz, $CDCl_3$) δ in ppm:188.41, 170.35, 162.61, 153.49, 148.18, 139.89, 135.95, 134.16, 134.05, 133.48, 131.55, 131.14, 131.03,128.99, 127.67, 127.44, 125.97, 125.87, 123.99, 123.87, 114.65, 66.61, 28.28; Anal. Calcd for $C_{25}H_{18}Cl_2N_2O_2S$: C, 62.37; H, 3.77; N, 5.82; Found: C, 62.54; H, 3.56; N, 5.46.

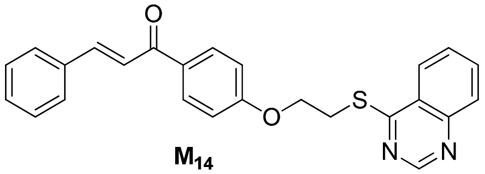

M14

(E)-3-phenyl-1-(4-(2-(quinazolin-4-ylthio)ethoxy)phenyl)prop-2-en-1-one (**M14**) White solid; m.p. 123.5–124.9 °C; yield, 62.7%; IR (KBr, cm^{-1}) v: 3035.1-3078.5 (C–H of benzene), 1662.7 (C=N), 1608.7 (C=O), 1485.2–1560.4 (C=C and benzene and Qu-ring), 1321.3 (C–N), 1254.7 (C–O); ^{1}H-NMR (500 MHz, $CDCl_3$) δ in ppm: 9.02 (s, 1H, Qu-2-H), 8.11 (d, J = 8.59 Hz, 1H, Qu-8-H), 8.07–8.04 (m, 3H, Ar–CH, CO–Ph-2,6-H), 7.99 (d, J = 8.59 Hz, 1H,Qu-5-H), 7.87 (t, J_1 = 8.59 Hz, J_2 = 6.87 Hz, 1H, Qu-7-H), 7.81 (dd, J_1 = J_2 = 5.73 Hz, 2H, Ar-2,6-H), 7.65–7.53 (m, 3H, Qu-6-H, Ar-6-H, Ph–CO=CH), 7.55 (dd, J_1 = 5.15 Hz, J_2 = 4.58 Hz, 1H, Ar-4-H), 7.42–7.40 (m, 2H, Ar-3,5-H), 7.08 (d, J = 9.16 Hz, 2H,CO–Ph-3,5-H), 4.43 (t, J_1 = 6.30 Hz, J_2 = 6.87 Hz, 2H, –OCH_2–), 3.82 (t, J_1 = 6.30 Hz, J_2 = 6.87 Hz, 2H, –SCH_2–); ^{13}C-NMR (125 MHz, $CDCl_3$) δ in ppm:188.79, 170.37, 162.37, 153.51, 148.17, 144.27, 144.19, 135.11, 134.05, 131.48, 130.48, 129.03, 128.99, 128.48, 127.66, 123.99, 123.88, 121.87,

114.56, 66.56, 28.30;Anal. Calcd for $C_{25}H_{20}N_2O_2S$: C, 72.79.; H, 4.89; N, 6.79; Found: C, 73.12; H, 5.11; N, 5.91.

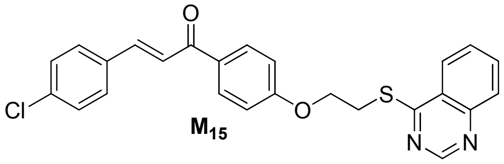

$\mathbf{M_{15}}$

(E)-3-(4-chlorophenyl)-1-(4-(2-(quinazolin-4-ylthio)ethoxy)phenyl)prop-2-en-1-one (**$\mathbf{M_{15}}$**) Yellow solid; m.p. 188.5–190.1 °C; yield, 57.7%; IR (KBr, cm^{-1}) *v*: 3042.8–3072.7 (C–H), 1662.7 (C=N), 1607.7 (C=O), 1485.2–1560.4 (C=C and benzene and Qu-ring), 1320.3 (C–N), 1257.6 (C–O); ^{1}H-NMR (500 MHz, $CDCl_3$) δ in ppm: 9.01 (s, 1H, Qu-2-H), 8.10 (d, *J* = 8.25 Hz, 1H,Qu-8-H), 8.04 (d, *J* = 9.20 Hz, 2H, CO–Ph-2,6-H), 7.99 (d, *J* = 8.25 Hz, 1H, Qu-5-H), 7.87 (t, J_1 = 8.20 Hz, J_2 = 7.35 Hz, 1H, Qu-7-H), 7.75 (d, *J* = 15.55 Hz, 1H, Ar–CH), 7.62 (t, J_1 = 6.85 Hz, J_2 = 8.25 Hz, 1H, Qu-6-H), 7.57 (d, *J* = 8.25 Hz, 2H,CO–Ph-3,5-H), 7.51 (d, *J* = 15.55 Hz, 1H, Ph–CO–CH), 7.39 (d, *J* = 8.25 Hz, 2H,Ar-2,6-H), 7.08 (d, *J* = 9.20 Hz, 2H, Ar-3,4-H), 4.43 (t, J_1 = J_2 = 6.30 Hz, 2H, $-OCH_2-$), 3.82 (t, J_1 = 6.40 Hz, J_2 = 6.90 Hz, 2H, $-SCH_2-$); ^{13}C-NMR (125 MHz, $CDCl_3$) δ in ppm:188.47, 170.34, 162.49, 153.49, 148.21, 142.64, 136.31, 134.02, 133.59, 133.65, 130.95, 129.58,129.29, 129.00, 127.64, 124.00, 123.86, 122.36, 114.62, 66.62, 28.30; Anal. Calcd for $C_{25}H_{19}ClN_2O_2S$: C, 67.18; H, 4.23; N, 6.27; Found: C, 67.63; H, 4.67; N, 6.26.

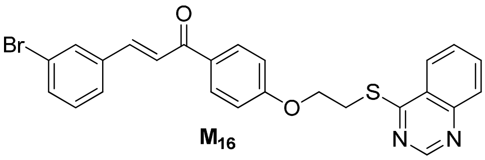

$\mathbf{M_{16}}$

(E)-3-(3-bromophenyl)-1-(4-(2-(quinazolin-4-ylthio)ethoxy)phenyl)prop-2-en-1-one (**$\mathbf{M_{16}}$**) Yellow solid; m.p. 124.5–125.8 °C; yield, 56.5%; IR (KBr, cm^{-1}) *v*: 3041.8–3079.4 (C–H of benzene), 1652.1 (C=N), 1602.9 (C=O), 1463.0–1564.3 (C=C and benzene and Qu-ring), 1326.1 (C–N), 1268.2 (C–O); ^{1}H-NMR (500 MHz, $CDCl_3$) δ in ppm: 9.02 (s, 1H, Qu-2-H), 8.11 (d, 1H, *J* = 9.20 Hz, Qu-8-H), 8.05 (d, 2H, *J* = 9.15 Hz, CO–Ph-2,6-H), 7.99 (s, 1H, Ar-2-H), *J* = 8.05 Hz, Qu-5-H), 7.89 (t, 1H, J_l = 8.60 Hz, J_2 = 6.85 Hz, Qu-7-H), 7.79 (s, 1H,) 7.72 (d, 1H, *J* = 16.05 Hz, Ar–CH), 7.61 (t, 1H, J_1 = 6.90 Hz, J_2 = 8.55 Hz, Qu-6-H), 7.54–7.51 (m, 3H, Ar-4,6-H, Ph–CO–CH), 7.29 (t, 1H, J_1 = J_2 = 8.00 Hz, Ar-5-H), 7.08 (d, 2H, *J* = 9.15 Hz, CO–Ph-3,5-H,), 4.43 (t, 2H, J_1 = J_2 = 6.60 Hz, $-OCH_2-$), 3.82 (t, 2H, J_1 = J_2 = 6.60 Hz, $-SCH_2-$); ^{13}C-NMR (125 MHz, $CDCl_3$) δ in ppm:188.29, 170.36, 162.56, 153.50, 148.17, 142.39, 137.25, 134.06, 133.17, 131.18, 131.03, 130.79, 130.53, 128.99, 127.67, 127.33, 123.99, 123.88, 123.14, 123.09, 114.62, 66.59, 28.28; Anal. Calcd for $C_{25}H_{19}BrN_2O_2S$: C, 61.10; H, 3.90; N, 5.70; Found: C, 61.52; H, 3.46; N, 5.31.

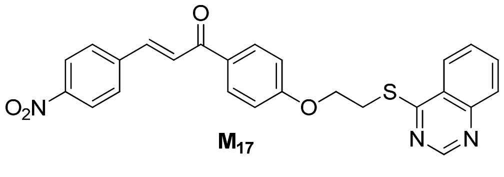

M17

(E)-3-(4-nitrophenyl)-1-(4-(2-(quinazolin-4-ylthio)ethoxy)phenyl)prop-2-en-1-one (**M17**) Yellow solid; m.p. 184.8–186.2 °C; yield, 61.3%; IR (KBr, cm^{-1}) *v*: 3041.8–3053.4 (C–H of benzene), 1663.6 (C=N), 1600.9 (C=O), 1485.2–1560.4 (C=C and benzene and Qu-ring), 1341.5 (C–N), 1260.5 (C–O); ^{1}H-NMR (500 MHz, $CDCl_3$) δ in ppm: 9.02 (s, 1H, Qu-2-H), 8.29 (d, *J* = 8.59 Hz, 2H, Ar-3,5-H), 8.11 (d, *J* = 8.59 Hz, 1H, Qu-8-H), 8.07 (d, *J* = 8.59 Hz, 2H, Ar-2,6-H), 7.99 (d, *J* = 8.02 Hz, 1H, Qu-5-H), 7.88 (t, J_1 = 8.59 Hz, J_2 = 6.87 Hz, 1H, Qu-7-H), 7.83–7.80 (m, 3H, Ar–CH, CO–Ph-3,5-H), 7.67 (d, *J* = 16.04 Hz, 1H, Ph–CO=CH), 7.62 (d, *J* = 8.02 Hz, 1H, Qu-6-H), 7.10 (d, *J* = 9.16 Hz, 2H, CO–Ph-3,5-H), 4.44 (t, J_1 = J_2 = 6.59 Hz, 2H, $–OCH_2–$), 3.83 (t, J_1 = J_2 = 6.30 Hz, 2H, $–SCH_2–$);^{13}C-NMR (125 MHz, $CDCl_3$) δ in ppm:187.83, 170.31, 162.83, 161.91, 153.49, 148.17, 141.33, 140.92, 134.08, 131.13, 129.92, 129.01, 128.95, 127.69, 126.32, 125.64, 124.31, 123.86, 114.75, 66.65, 28.25; Anal. Calcd for $C_{25}H_{19}N_3O_4S$: C, 65.63; H, 4.19; N, 9.18; Found: C, 66.02; H, 3.87; N, 8.75.

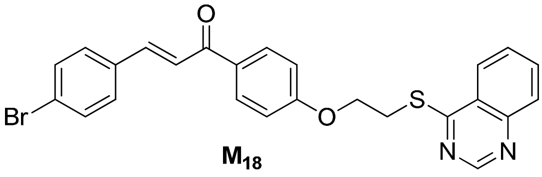

M18

(E)-3-(4-bromophenyl)-1-(4-(2-(quinazolin-4-ylthio)ethoxy)phenyl)prop-2-en-1-one (**M18**) Yellow solid; m.p. 204.2–205.7 °C; yield, 56.1%; IR (KBr, cm^{-1}) *v*: 3042.8-3073.7 (C–H of benzene), 1654.0 (C=N), 1604.8 (C=O), 1483.6–1560.4 (C=C and benzene and Qu-ring), 1319.3 (C–N), 1253.7 (C–O); ^{1}H-NMR (500 MHz, $CDCl_3$) δ in ppm: 9.01 (s, 1H, Qu-2-H), 8.09 (d, *J* = 7.45 Hz, 1H, Qu-8-H), 8.04 (d, *J* = 9.15 Hz, 2H, CO–Ph-2,6-H), 7.98 (d, *J* = 8.60 Hz, 1H, Qu-5-H), 7.87 (t, J_1 = 8.60 Hz, J_2 = 6.85 Hz, 1H, Qu-7-H), 7.74 (d, *J* = 16.00 Hz, 1H, Ar–CH), 7.60 (t, J_1 = 8.05 Hz, J_2 = 7.40 Hz, 1H, Qu-6-H), 7.54–7.48 (m, 5H, Ar-2,3,5,6-H, Ph–CO–CH), 7.07 (d, *J* = 8.60 Hz, 2H, CO–Ph-3,5-H,), 4.42 (t, J_1 = 6.85 Hz, J_2 = 6.30 Hz, 2H, $–OCH_2–$), 3.81 (t, J_1 = 6.75 Hz, J_2 = 6.30 Hz, 2H, $–SCH_2–$); ^{13}C-NMR (125 MHz, $CDCl_3$) δ in ppm:188.39, 170.33, 162.49, 153.50, 148.16, 142.71, 134.05, 134.03, 132.24, 131.26, 130.97, 129.83,128.99, 127.66, 124.68, 123.97, 123.87, 122.33, 114.60, 66.57, 28.27; Anal. Calcd for $C_{25}H_{19}BrN_2O_2S$: C, 61.10; H, 3.90; N, 5.70; Found: C, 61.43; H, 3.42; N, 5.18.

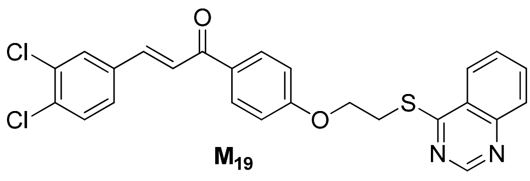

M19

(E)-3-(3,4-dichlorophenyl)-1-(4-(2-(quinazolin-4-ylthio)ethoxy)phenyl)prop-2-en-1-one (**M19**) Yellow solid; m.p. 107.1–108.9 °C; yield, 58.3%; IR (KBr, cm^{-1}) *v*: 3047.6–3074.6 (C–H of benzene), 1662.7 (C=N), 1609.6 (C=O), 1485.2–1560.4 (C=C and benzene and Qu-ring), 1331.9 (C–N), 1255.7 (C–O); ^{1}H-NMR (500 MHz, $CDCl_3$) δ: 9.02 (s, 1H, Qu-2-H), 8.11 (d, *J* = 8.02 Hz, 1H, Qu-8-H), 8.04 (d, *J* = 8.59 Hz, 2H, CO–Ph-2,6-H), 7.99 (d, *J* = 8.02 Hz, 1H, Qu-5-H), 7.88 (t, J_1 = 8.59 Hz, J_2 = 6.87 Hz,

1H, Qu-7-H), 7.72–7.66 (m, 2H, Ar–CH, Ar-6-H), 7.61 (t, J_1 = 8.02 Hz, J_2 = 7.45 Hz, 1H, Qu-6-H), 7.53–7.44 (m, 3H, Ph–CO=CH, Ar-2,5-H), 7.09 (d, J = 9.16 Hz, 2H, CO–Ph-3,5-H), 4.43 (t, J_1 = 6.87 Hz, J_2 = 6.30 Hz, 2H, –OCH2–), 3.82 (t, J_1 = J_2 = 6.59 Hz, 2H, –SCH2–); ^{13}C-NMR (125 MHz, CDCl3) δ: 188.05, 170.38, 162.63, 153.48,148.13, 141.27, 135.19, 134.26, 134.08, 133.34, 132.26, 131.07, 131.03, 129.73, 128.97, 127.69, 127.60, 123.98, 123.87, 123.35, 114.66, 66.60, 28.28; Anal. Calcd for $C_{25}H_{18}Cl_2N_2O_2S$: C, 62.37; H, 3.77; N, 5.82; Found: C, 62.01; H, 3.93; N, 6.07.

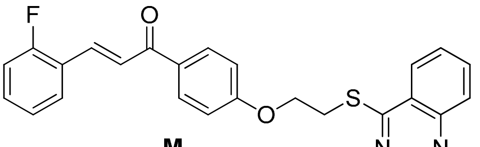

M20

(E)-3-(2-fluorophenyl)-1-(4-(2-(quinazolin-4-ylthio)ethoxy)phenyl)prop-2-en-1-one (**M20**) Yellow solid; m.p. 118.0–119.8 °C; yield, 58.3%; IR (KBr, cm^{-1}) *v*: 3032.2–3078.2 (C–H of benzene), 1661.8 (C=N), 1609.2 (C=O), 1463.4–1561.4 (C=C and benzene and Qu-ring), 1324.1 (C–N), 1252.2 (C–O); ^{1}H-NMR (500 MHz, CDCl3) δ in ppm: 9.02 (s, 1H, Qu-2-H), 8.11 (d, J = 8.02 Hz, 1H,Qu-8-H), 8.06 (d, J = 9.16 Hz, 2H, CO–Ph-2,6-H), 7.99 (d, J = 8.59 Hz, 1H, Qu-5-H), 7.90–7.86 (m, 2H, Ar–CH, Qu-7-H), 7.67–7.59 (d, 3H,Ph–CO=CH, Qu-6-H, Ar-4-H), 7.38 (d, 1H, J = 8.02 Hz, 1H, Ar-6-H), 7.19 (t, J_1 = 7.45 Hz, J_2 = 6.87 Hz, 1H, Ar-3-H), 7.13 (t, J_1 = 8.59 Hz, J_2 = 8.02 Hz, 1H, Ar-5-H), 7.08 (d, J = 8.59 Hz, 2H, CO–Ph-3,5-H), 4.42 (t, J_1 = J_2 = 6.30 Hz, 2H, –OCH2–), 3.82 (t, J_1 = 6.30 Hz, J_2 = 6.87 Hz, 2H, –SCH2–); ^{13}C-NMR (125 MHz, CDCl3) δ in ppm: 188.78, 170.38, 162.46, 153.49, 148.17, 136.94, 134.05, 131.77, 131.69, 131.32, 131.05, 129.92, 128.98, 127.66, 124.57, 124.52, 123.91, 123.19, 116.47, 116.29, 114.59, 66.57, 28.30; Anal. Calcd for $C_{25}H_{19}FN_2O_2S$: C, 69.75; H, 4.45; N, 6.51; Found: C, 70.19; H, 4.02; N, 6.12.

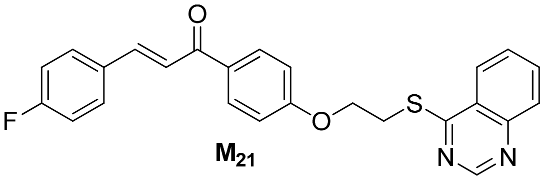

M21

(E)-3-(4-fluorophenyl)-1-(4-(2-(quinazolin-4-ylthio)ethoxy)phenyl)prop-2-en-1-one (**M21**) Yellow solid; m.p. 178.5–180.1 °C; yield, 59.1%; IR (KBr, cm^{-1}) *v*: 3046.7–3074.6 (C–H of benzene), 1663.6 (C=N), 1609.6 (C=O), 1485.2–1561.4 (C=C and benzene and Qu-ring), 1321.3 (C–N), 1255.7 (C–O); ^{1}H-NMR (500 MHz, CDCl3) δ in ppm: 9.01 (s, 1H, Qu-2-H), 8.10 (d, J = 8.55 Hz, 1H, Qu-8-H), 8.04 (d, J = 9.15 Hz, 2H, CO–Ph-2,6-H), 7.99 (d, J = 8.00 Hz, 1H, Qu-5-H), 7.87 (t, J_1 = 7.45 Hz, J_2 = 8.05 Hz, 1H, Qu-7-H), 7.77 (d, J = 15.45 Hz, 1H, Ar–CH), 7.64–7.59 (m, 3H, Qu-6-H, Ar-2,6-H), 7.47 (d, J = 15.46 Hz, 1H, Ph–CO–CH), 7.10 (t, J_1 = J_2 = 8.59 Hz, 2H, Ar-3,5-H), 7.07 (d, J = 9.16 Hz, 2H, CO–Ph-3,5-H), 4.42 (t, J_1 = 6.85 Hz, J_2 = 6.30 Hz, 2H, –OCH2–), 3.82 (t, J_1 = 6.85 Hz, J_2 = 6.30 Hz, 2H, –SCH2–); ^{13}C-NMR (125 MHz, CDCl3) δ in ppm: 188.53, 170.35, 162.45, 153.49, 148.21, 142.83, 134.02, 131.43, 130.92, 130.34, 130.28,128.99, 127.64, 124.00, 123.86, 121.64, 116.25, 116.07, 114.60, 66.61, 28.31; Anal. Calcd for $C_{25}H_{19}FN_2O_2S$: C, 69.75; H, 4.45; N, 6.51; Found: C, 69.66; H, 4.42; N, 6.46.

References

1. Liu, G.; Song, B.A.; Sang, W.J.; Yang, S.; Jin, L.H.; Ding, X. Synthesis and bioactivity of *N*-aryl-4-aminoquinazoline compounds. *Chin. J. Org. Chem.* **2004**, *10*, 1296–1299.
2. Karminski, W.; Kulicka, J.; Miernik, J.W. The synthesis of some quinazoline derivatives and their biological properties. *J. Environ. Sci. Health* **1983**, *18*, 599–610.
3. Zhao, P.L.; Liu, C.L.; Huang, W.; Wang, Y.Z.; Yang, G.F. Synthesis and fungicidal evaluation of novel chalcone-based strobilurin analogues. *J. Agric. Food Chem.* **2007**, *55*, 5697–5700.
